# Multiomic neuropathology improves diagnostic accuracy in pediatric neuro-oncology

**DOI:** 10.1038/s41591-023-02255-1

**Published:** 2023-03-16

**Authors:** Dominik Sturm, David Capper, Felipe Andreiuolo, Marco Gessi, Christian Kölsche, Annekathrin Reinhardt, Philipp Sievers, Annika K. Wefers, Azadeh Ebrahimi, Abigail K. Suwala, Gerrit H. Gielen, Martin Sill, Daniel Schrimpf, Damian Stichel, Volker Hovestadt, Bjarne Daenekas, Agata Rode, Stefan Hamelmann, Christopher Previti, Natalie Jäger, Ivo Buchhalter, Mirjam Blattner-Johnson, Barbara C. Jones, Monika Warmuth-Metz, Brigitte Bison, Kerstin Grund, Christian Sutter, Steffen Hirsch, Nicola Dikow, Martin Hasselblatt, Ulrich Schüller, Arend Koch, Nicolas U. Gerber, Christine L. White, Molly K. Buntine, Kathryn Kinross, Elizabeth M. Algar, Jordan R. Hansford, Nicholas G. Gottardo, Martin U. Schuhmann, Ulrich W. Thomale, Pablo Hernáiz Driever, Astrid Gnekow, Olaf Witt, Hermann L. Müller, Gabriele Calaminus, Gudrun Fleischhack, Uwe Kordes, Martin Mynarek, Stefan Rutkowski, Michael C. Frühwald, Christof M. Kramm, Andreas von Deimling, Torsten Pietsch, Felix Sahm, Stefan M. Pfister, David. T. W. Jones

**Affiliations:** 1 Hopp Children’s Cancer Center Heidelberg (KiTZ), Heidelberg, Germany; 2 Division of Pediatric Glioma Research, German Cancer Research Center (DKFZ) and German Cancer Consortium (DKTK), Heidelberg, Germany; 3 Department of Pediatric Oncology, Hematology & Immunology, Heidelberg University Hospital, Heidelberg, Germany; 4 Department of Neuropathology, Charité - Universitätsmedizin Berlin, corporate member of Freie Universität Berlin and Humboldt-Universität zu Berlin, Berlin, Germany; 5 German Cancer Consortium (DKTK), Partner Site Berlin, German Cancer Research Center (DKFZ), Heidelberg, Germany; 6 Department of Neuropathology, DGNN Brain Tumor Reference Center, University of Bonn, Bonn, Germany; 7 Laboratory of Neuropathology, Paulo Niemeyer State Brain Institute, Rio de Janeiro, Brazil; 8 D’Or Institute for Research and Education (IDOR), Rio de Janeiro, Brazil; 9 Institute of Pathology, Heidelberg University Hospital, Heidelberg, Germany; 10 Department of Neuropathology, Heidelberg University Hospital, Heidelberg, Germany; 11 Institute of Neuropathology, University Medical Center Hamburg-Eppendorf, Hamburg, Germany; 12 Clinical Cooperation Unit Neuropathology, German Cancer Research Center (DKFZ) and German Cancer Consortium (DKTK), Heidelberg, Germany; 13 Department of Neurological Surgery, Helen Diller Research Center, University of California, San Francisco, San Francisco, CA USA; 14 Division of Pediatric Neurooncology, German Cancer Research Center (DKFZ) and German Cancer Consortium (DKTK), Heidelberg, Germany; 15 Department of Pediatric Oncology, Dana-Farber Cancer Institute, Boston, MA USA; 16 Broad Institute of MIT and Harvard, Cambridge, MA USA; 17 Omics IT and Data Management Core Facility, German Cancer Research Center (DKFZ) and German Cancer Consortium (DKTK), Heidelberg, Germany; 18 Department of Diagnostic and Interventional Neuroradiology, University Hospital of Würzburg, Würzburg, Germany; 19 Neuroradiological Reference Center for the Pediatric Brain Tumor (HIT) Studies of the German Society of Pediatric Oncology and Hematology, University Hospital Würzburg, since 2021 University Hospital Augsburg, Augsburg, Germany; 20 Diagnostic and Interventional Neuroradiology, Faculty of Medicine, University of Augsburg, Augsburg, Germany; 21 Institute of Human Genetics, Heidelberg University Hospital, Heidelberg, Germany; 22 Institute of Neuropathology, University Hospital Münster, Münster, Germany; 23 Department of Paediatric Haematology and Oncology, University Medical Center Hamburg-Eppendorf, Hamburg, Germany; 24 Research Institute Children’s Cancer Center Hamburg, Hamburg, Germany; 25 Department of Oncology, University Children’s Hospital Zürich, Zürich, Switzerland; 26 Genetics and Molecular Pathology Laboratory, Hudson Institute of Medical Research, Clayton, VIC Australia; 27 Department of Molecular and Translational Science, Monash University, Melbourne, VIC Australia; 28 Victorian Clinical Genetics Services, Parkville, VIC Australia; 29 Australian and New Zealand Children’s Haematology and Oncology Group (ANZCHOG), Hudson Institute of Medical Research, Clayton, VIC Australia; 30 Department of Paediatrics, University of Melbourne, Parkville, VIC Australia; 31 Women’s and Children’s Hospital, South Australia Health and Medical Research Institute, South Australia immunoGENomics Cancer Institute, University of Adelaide, Adelaide, SA Australia; 32 Department of Paediatric and Adolescent Oncology/Haematology, Perth Children’s Hospital, Nedlands, WA Australia; 33 Centre for Child Health Research, University of Western Australia, Nedlands, WA Australia; 34 Brain Tumour Research Program, Telethon Kids Institute, Nedlands, WA Australia; 35 Department of Neurosurgery, University of Tuebingen, Tuebingen, Germany; 36 Department of Neurosurgery, Charité – Universitätsmedizin Berlin, Berlin, Germany; 37 German HIT-LOGGIC Registry for low-grade glioma in children and adolescents, Department of Pediatric Oncology and Hematology, Charité - Universitätsmedizin Berlin, corporate member of Freie Universität Berlin and Humboldt-Universität zu Berlin, Berlin, Germany; 38 Swabian Children’s Cancer Center, Paediatric and Adolescent Medicine, Faculty of Medicine, University Augsburg, Augsburg, Germany; 39 Clinical Cooperation Unit Pediatric Oncology, German Cancer Research Center (DKFZ) and German Cancer Consortium (DKTK), Heidelberg, Germany; 40 Department of Pediatrics and Pediatric Hematology/Oncology, University Children’s Hospital, Klinikum Oldenburg AöR, Oldenburg, Germany; 41 Department of Pediatric Hematology and Oncology, University Childrens’ Hospital Muenster, Muenster, Germany; 42 Pediatric Hematology and Oncology, Pediatrics III, University Children’s Hospital of Essen, Essen, Germany; 43 Mildred Scheel Cancer Career Center HaTriCS4, University Medical Center Hamburg-Eppendorf, Hamburg, Germany; 44 Department of Child and Adolescent Health, Division of Pediatric Hematology and Oncology, University Medical Center Göttingen, Göttingen, Germany

**Keywords:** CNS cancer, Paediatric cancer, Cancer epigenetics, Cancer epidemiology, Diagnostic markers

## Abstract

The large diversity of central nervous system (CNS) tumor types in children and adolescents results in disparate patient outcomes and renders accurate diagnosis challenging. In this study, we prospectively integrated DNA methylation profiling and targeted gene panel sequencing with blinded neuropathological reference diagnostics for a population-based cohort of more than 1,200 newly diagnosed pediatric patients with CNS tumors, to assess their utility in routine neuropathology. We show that the multi-omic integration increased diagnostic accuracy in a substantial proportion of patients through annotation to a refining DNA methylation class (50%), detection of diagnostic or therapeutically relevant genetic alterations (47%) or identification of cancer predisposition syndromes (10%). Discrepant results by neuropathological WHO-based and DNA methylation-based classification (30%) were enriched in histological high-grade gliomas, implicating relevance for current clinical patient management in 5% of all patients. Follow-up (median 2.5 years) suggests improved survival for patients with histological high-grade gliomas displaying lower-grade molecular profiles. These results provide preliminary evidence of the utility of integrating multi-omics in neuropathology for pediatric neuro-oncology.

## Abstract (web summary)

The integration of DNA methylation profiling and targeted sequencing with neuropathology improves the diagnostic accuracy of central nervous system tumors in a population-based cohort of more than 1,200 newly diagnosed pediatric patients.

## Main

Children and adolescents can be diagnosed with a broad spectrum of central nervous system (CNS) tumors with divergent clinical behavior. The recently updated World Health Organization (WHO) classification of CNS tumors^1,2^ recognizes a plethora of variants that can be difficult to distinguish. Some are exceedingly rare, such that a neuropathologist would see only very few cases over the course of their career. To improve diagnostic accuracy in neuro-oncology, we developed a neuro-oncology-specific next-generation sequencing (NGS) gene panel^3^ and introduced a DNA methylation-based classification system for CNS tumors^4^. Since 2016, the accompanying online research tool for CNS tumor classification from DNA methylation data has seen more than 90,000 sample uploads. Although the benefit of implementing this tool in specialized centers has been reported—especially for difficult-to-diagnose tumors^5–7^—its utility in a routine diagnostic setting still has to be evaluated. We launched the Molecular Neuropathology 2.0 (MNP 2.0) study as part of the German pediatric neuro-oncology ‘Treatment Network HIT’, aiming to integrate DNA methylation analysis and gene panel sequencing with blinded central neuropathological assessment for a population-based cohort of pediatric patients with CNS tumors at the time of primary diagnosis.

## Results

### Patient recruitment and sample processing

**Fig. 1:**
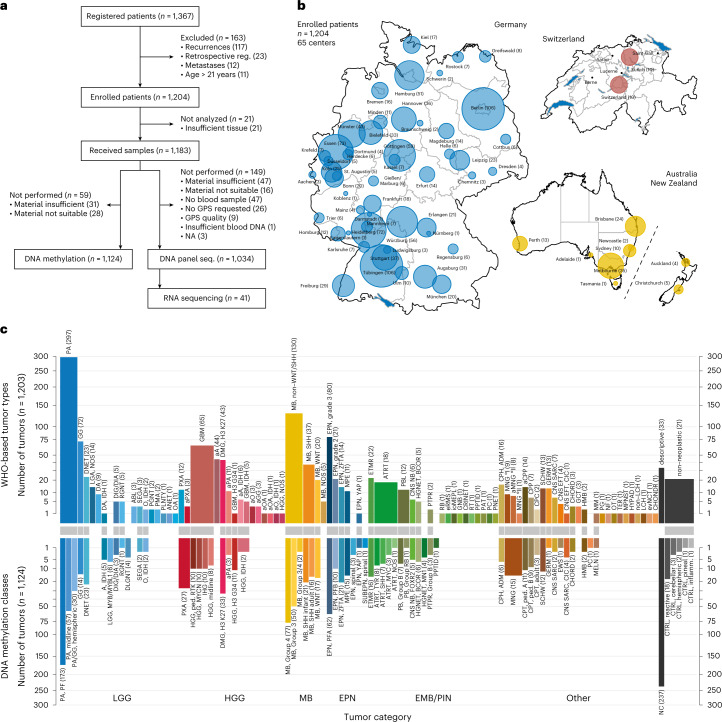
Study design, patient recruitment and CNS tumor classification. **a**, CONSORT flow diagram for 1,367 patients registered between April 2015 and March 2019. **b**, Schematic geographical overview of 1,204 enrolled patients by center of recruitment. Circle size is proportional to the number of patients. Country size is not to scale. **c**, Tumor classification into WHO-based CNS tumor types (upper panel) and DNA methylation classes (lower panel). Numbers in brackets indicate tumors per tumor type or class. Corresponding and overlapping tumor types and classes are indicated by connecting gray bars. *y*-axis scale is square root transformed for improved visibility of tumor types and classes occurring at low frequency. See Extended Data Fig. 1 for a full list of individual abbreviations. See Supplementary Table 1 for underlying data. GPS, gene panel sequencing.

Over a 4-year period (April 2015 to March 2019), 1,204 patients with available formalin-fixed, paraffin-embedded (FFPE) tumor tissue were enrolled, excluding 163 patients who did not fulfill the inclusion criteria (117 recurrences, 23 retrospective registrations, 12 metastases, 11 adults) (Fig. 1a). Patients were enrolled from 65 centers in Germany, Australia/New Zealand (starting June 2017) and Switzerland (starting July 2017) in a population-based manner (Fig. 1b and Supplementary Figs. 1 and 2). In 59 tumors, received tissue was either insufficient (31, 2.6%) or not suitable (28, 2.4%) for DNA methylation analysis and/or NGS (4.0% and 1.4%, respectively) (Fig. 1a). Median time from arrival of FFPE sections at the molecular testing laboratory to first molecular report was 21 days (Supplementary Fig. 3a,b). Timelines from tumor surgery to successful patient registration were shorter in centers with higher recruitment rates (Supplementary Fig. 3c).

### CNS tumor classification

#### WHO-based CNS tumor types by neuropathological assessment

**Extended Data Fig. 1:**
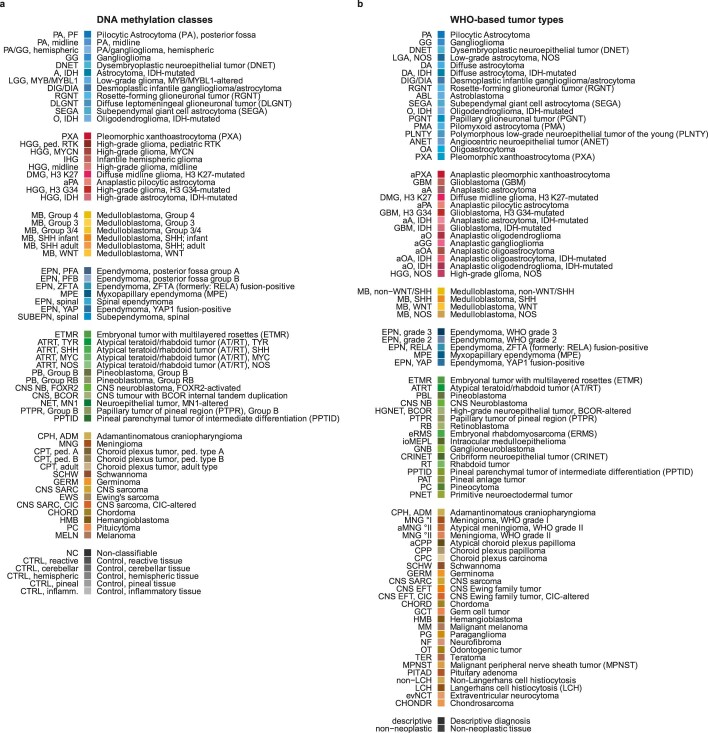
Tumor class and type color legend and abbreviations. **a**, DNA methylation classes, abbreviations and colors used for representation in this article. **b**, WHO-based diagnoses, abbreviations and colors used for representation in this article. Corresponding DNA methylation classes and WHO-based diagnoses share the same color hue; overlapping DNA methylation classes and WHO-based diagnoses share shades of the same color hue. DNA methylation classes and WHO-based diagnoses from the same tumor category share a similar color hue spectrum.

**Extended Data Fig. 2:**
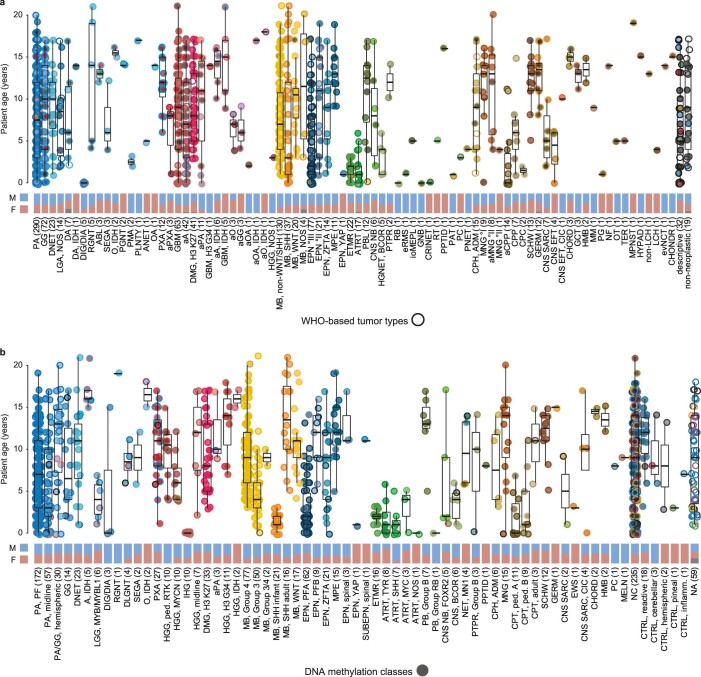
Clinical patient characteristics. Patient age (combined scattered and boxplots, upper panel) and sex (stacked bar charts, lower panel) across WHO-based diagnoses (**a**) and DNA methylation classes (**b**). Each tumor is represented by a circle indicating assigned WHO-based tumor type (outline) and DNA methylation class (fill), and colors correspond to tumor types and classes as indicated in Fig. 1 and Extended Data Fig. 1. Numbers in brackets indicate tumors per tumor class or type with available data. Center line, median; box limits, upper and lower quartiles; whiskers, 1.5 x interquartile range. F, female; M, male. See Supplementary Table 1 for underlying data.

The distribution of tumor types by reference neuropathological evaluation according to the WHO classification of CNS tumors, and the corresponding clinical patient data, were considered representative of a population-based cohort of pediatric patients with CNS tumors undergoing tumor biopsy or resection (Fig. 1c, Extended Data Figs. 1 and 2a, Supplementary Fig. 2 and Supplementary Table 1). Comparison with epidemiological data from the German Childhood Cancer Registry^8^ showed an annual recruitment of up to 64% of all patients newly diagnosed with CNS tumors (Supplementary Fig. 2b). Neurofibromatosis type 1-associated or diffuse midline gliomas may have been underrepresented, as they are not consistently biopsied. No neoplastic tissue was detected in 21 samples (1.7%). In the remaining 1,182 tumors, a confident diagnosis was assigned in 1,028 cases (87.0%), whereas 77 were compatible with and 22 suspicious of a certain tumor type (6.5% and 1.9%, respectively). A descriptive diagnosis was established for 55 tumors (4.7%), including 33 (2.7%) that could not be assigned to any tumor category. The most common diagnostic categories were low-grade glial/glioneuronal (LGG) tumors (37.7%), medulloblastomas (MBs, 16.0%), high-grade gliomas (HGGs, 15.6%), ependymal (EPN) tumors (10.6%) and other embryonal or pineal (EMB/PIN) tumors (6.2%) (Supplementary Fig. 2a). Various other less frequent tumor types made up a total of 9.5% of the cohort. Patient age and sex were distributed as expected (Extended Data Fig. 2a).

#### DNA methylation-based CNS tumor classification

**Extended Data Fig. 3:**
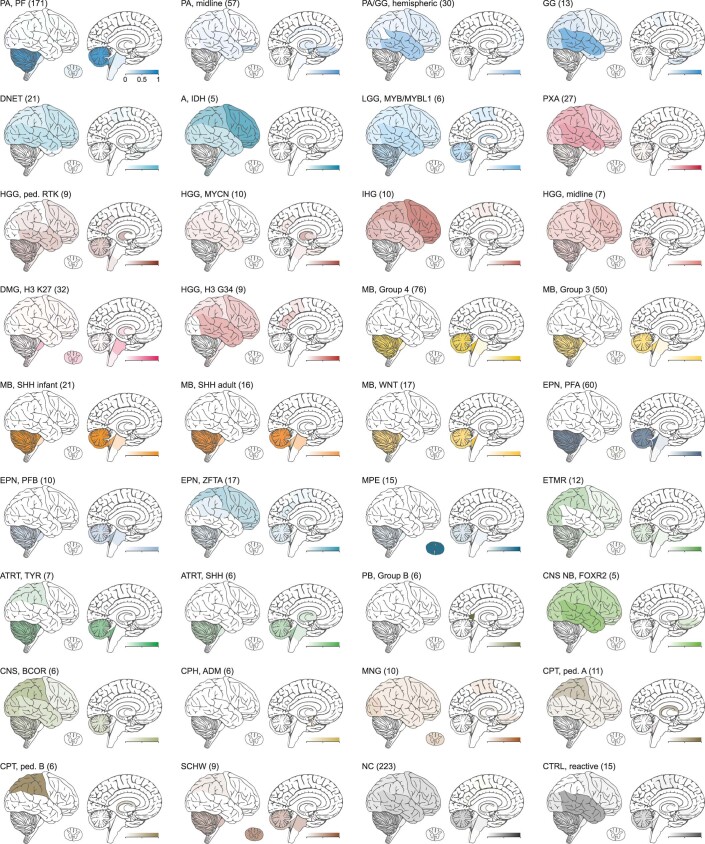
Tumor location by DNA methylation class. Heatmap representation of tumor location by DNA methylation class for classes with ≥ five samples. Numbers in brackets indicate tumors with available data. Color scales indicate the fraction of tumors affecting an anatomical region, and colors correspond to tumor types and classes as indicated in Fig. 1 and Extended Data Fig. 1. See Supplementary Table 1 for underlying data.

**Extended Data Fig. 4:**
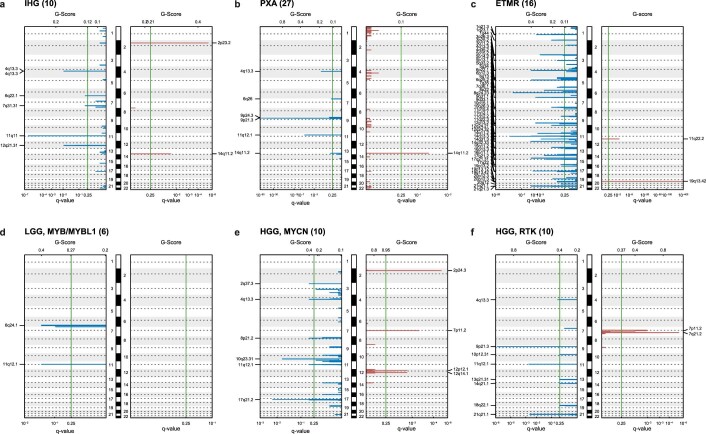
Significant regions of DNA copy number alterations by DNA methylation class. Plots show the q-values (x-axes, indicating the false discovery rate) determined by GISTIC2.0 with respect to significant lost (blue) and gained (red) genomic regions among the human chromosomes 1 to 22 (hg19) in DNA methylation classes ‘infantile hemispheric glioma’ (**a**), ‘pleomorphic xanthoastrocytoma’ (**b**), ‘embryonal tumor with multilayered rosettes’ (ETMR, **c**), ‘low-grade glioma, MYB/MYBL1-altered’ (**d**), ‘high-grade glioma, MYCN’ (**e**), and ‘high-grade glioma, pediatric RTK’ (**f**). Numbers in brackets indicate sample size for each class. Green lines indicate the significance threshold of q-value < 0.25. The cytobands of significantly altered regions are denoted on the y-axes. See Supplementary Table 2 for a detailed overview of significantly amplified/deleted regions across all DNA methylation classes.

Using, in each case, the latest applicable version at the time of diagnosis (version 9.0–version 11b4; Methods (ref. ^4^)) of a DNA methylation-based random forest (RF) class prediction algorithm, tumors were assigned to 65 (from a possible 91) different DNA methylation classes (Fig. 1c, Extended Data Fig. 1 and Supplementary Table 1). Besides LGG (28.5%), MB (16.3%) formed the second largest category, followed by HGG (10.1%), EPN (10.1%) and other EMB/PIN tumors (5.5%), whereas the remaining 6.2% were distributed among other less frequent classes (Fig. 1c and Supplementary Fig. 2a). A substantial fraction of tumors (21.1%) could not be confidently assigned to a DNA methylation class. The DNA methylation profiles of 25 (2.2%) samples assigned to a control class of non-neoplastic tissue were indicative of low tumor cell content in the analyzed tissue. DNA methylation classes were associated with patterns of patient age, sex and tumor location (Extended Data Figs. 2b and 3) as well as DNA copy number alterations (Extended Data Fig. 4, Supplementary Figs. 4 and 5 and Supplementary Table 2). As examples, the DNA methylation class ‘infantile hemispheric glioma’ exclusively comprised hemispheric tumors in infants with frequent focal amplifications on cytoband 2p23.2, indicative of fusions involving the *ALK* gene^9,10^; the DNA methylation class ‘PXA’ comprised hemispheric tumors across ages consistently harboring homozygous deletions of the *CDKN2A/B* locus (9p21.3); and the DNA methylation class ‘ETMR’ comprised predominantly occipital or posterior fossa tumors in young children with a pathognomonic amplification at 19q13.42 (ref. ^11^). Additional significant copy number alterations included focal deletion involving the *MYB* locus in ‘LGG, MYB/MYBL1’ (6q24.1), amplification of *MYCN* in ‘HGG, MYCN’ (2p24.3) and amplification involving *EGFR* in ‘HGG, RTK’ (7p11.2) (Extended Data Fig. 4).

#### Comparison of WHO-based and DNA methylation-based classification

**Fig. 2:**
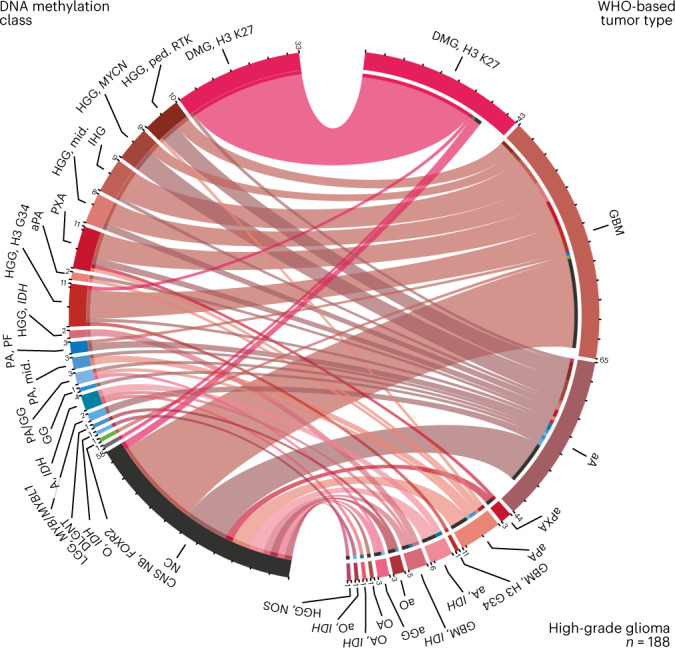
DNA methylation classes in WHO-based pediatric HGGs. Comparison of assigned DNA methylation classes (left semicircle) and WHO-based tumor types (right semicircle) across HGGs. Colors correspond to tumor types and classes as indicated in Fig. 1 and Extended Data Fig. 1. This category of HGGs is composed of WHO-based tumor type. See Supplementary Fig. 6 for composition by DNA methylation class. See Supplementary Table 1 for underlying data.

**Fig. 3:**
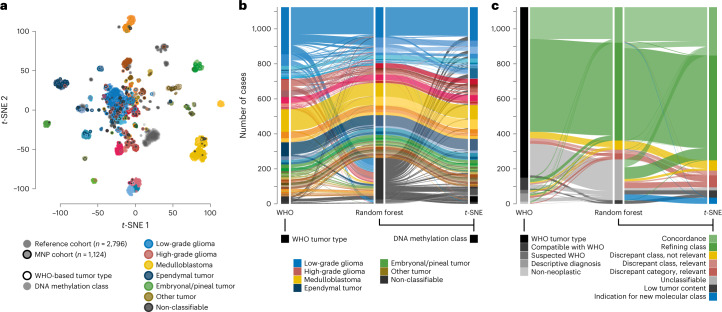
Landscape of DNA methylation classes and levels of concordance with WHO-based diagnosis. **a**, *t*-SNE analysis of DNA methylation data from the study cohort alongside 89 published DNA methylation classes^4^. Each tumor from the study cohort is represented by a circle indicating assigned DNA methylation class (fill) and WHO-based tumor type (outline). **b**, Comparison of WHO-based tumor types and DNA methylation classes assigned by RF-based class prediction and *t*-SNE analysis. Colors in **a** and **b** correspond to tumor types and classes as indicated in Fig. 1 and Extended Data Fig. 1. **c**, Comparison of certainty levels of WHO-based diagnoses and concordance with DNA methylation classes assigned by RF-based class prediction and *t*-SNE analysis. See Supplementary Tables 1 and 4 for underlying data.

**Extended Data Fig. 5:**
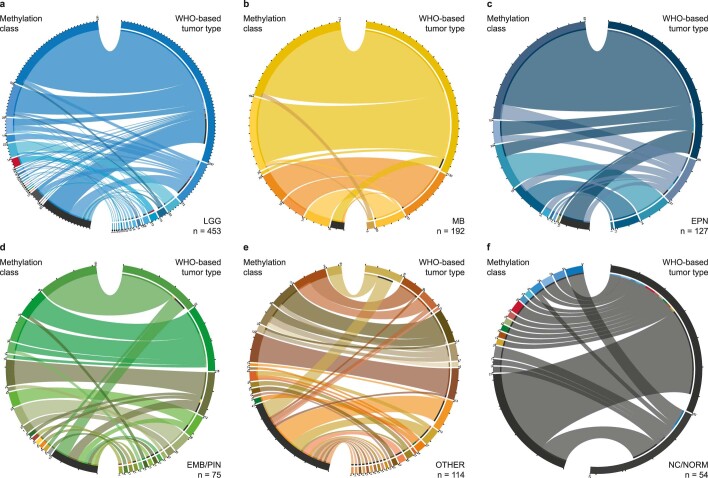
Comparison of WHO-based and DNA methylation-based tumor classification. Comparison of assigned DNA methylation classes (left semicircle) and WHO-based tumor types (right semicircle) across low-grade gliomas (LGG, **a**), medulloblastomas (MB, **b**), ependymal tumors (EPN, **c**), embryonal/pineal tumors (EMB/PIN, **d**), other types (**e**), and samples with a descriptive diagnosis or non-neoplastic tissue (**f**). Colors correspond to tumor types and classes as indicated in Fig. 1 and Extended Data Fig. 1. Categories in **a–f** are composed by WHO-based tumor type; see Supplementary Fig. 6 for composition by DNA methylation class. See Supplementary Table 1 for underlying data.

**Extended Data Fig. 6:**
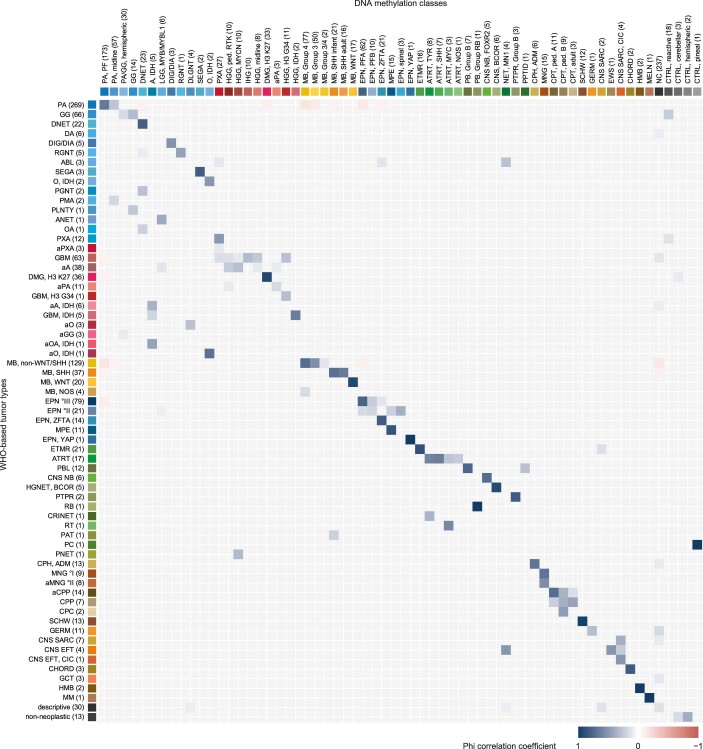
Correlation between DNA methylation-based and WHO-based CNS tumor classification. Correlation between DNA methylation-based and WHO-based CNS tumor classification. Phi correlation coefficient between DNA methylation-based classes and WHO-based tumor types is represented by a color scale as indicated. Numbers in brackets indicate the number of tumors per tumor type/class. Only correlations with a *P*-value < 0.01 are displayed; see Supplementary Fig. 7 for all possible correlations between DNA methylation classes and WHO-based tumor types. See Supplementary Table 1 and Supplementary Table 3 for underlying data.

Directly juxtaposing WHO-based tumor type and DNA methylation class for individual tumors (Fig. 2, Extended Data Fig. 5, Supplementary Fig. 6 and Supplementary Table 1) as well as pairwise comparison indicated strong correlation between combinations known to correspond or overlap across categories (Extended Data Fig. 6, Supplementary Fig. 7 and Supplementary Table 3) but also a high fraction of tumors unclassifiable by RF-based prediction among WHO-defined HGG (33.5%), LGG (20%) and other rare tumors (37.6%) (Fig. 2, Extended Data Fig. 5 and Supplementary Table 1). Visualization of DNA methylation patterns by *t*-distributed stochastic neighbor embedding (*t*-SNE) (Fig. 3a and Supplementary Table 4), and subsequent class assignment by visual inspection (Supplementary Fig. 8a), allowed classification of another 229 samples, with profiles of 34 tumors (3.0%) suggestive of novel molecular classes not represented in the original reference cohort^4^, such as HGG of the posterior fossa and neuroepithelial tumors with *PATZ1* fusions^12^ or *PLAGL1* fusions^13^ (Fig. 3b,c and Supplementary Fig. 8c). In most tumors (67.8%), neuropathological WHO-based tumor typing and DNA methylation class prediction were considered concordant, with an additional refinement by DNA methylation class in 49.7% of all cases (Fig. 3c and Supplementary Table 1). Assignments to a discrepant tumor class (within a category, 2.0%) or to a discrepant tumor category (3.0%) were considered clinically relevant (that is, changing the recommended treatment protocol) in 5% of all cases. This included 15 of 43 samples with inconclusive histology or no detectable tumor tissue, of which most (11/15) were classified as lower-grade glial or glioneuronal tumors by DNA methylation analysis (Extended Data Fig. 5f). There was an enrichment of clinically relevant discrepancies in histologically classified HGG (24/173, 13.9%) compared to other WHO-defined categories (*P* < 0.001). Among those, the most common combinations (21/24) included anaplastic (pilocytic) astrocytomas or glioblastomas (WHO grade 3–4) assigned to DNA methylation classes of lower-grade gliomas, including PA, GG or *MYB*/*MYBL*-altered tumors (WHO grade 1–2) (Fig. 2 and Supplementary Fig. 6a). Clinically relevant discrepancies were rarer in LGG (2.2%), MB (1.1%), EPN (1.6%) and other tumor types (0.0%). Discrepant tumor types and classes currently not considered clinically relevant were assigned in 4.6% of samples, affecting mostly lower-grade glial and glioneuronal tumors (29/52) (Extended Data Fig. 5a and Supplementary Fig. 6b). Samples could not be assigned to any tumor category or did not contain detectable tumor tissue by both neuropathological assessment and DNA methylation analysis in 1.4% and 0.7%, respectively (Extended Data Fig. 5f and Fig. 3b,c).

### Integration of NGS

#### Detection of relevant genetic alterations

**Fig. 4:**
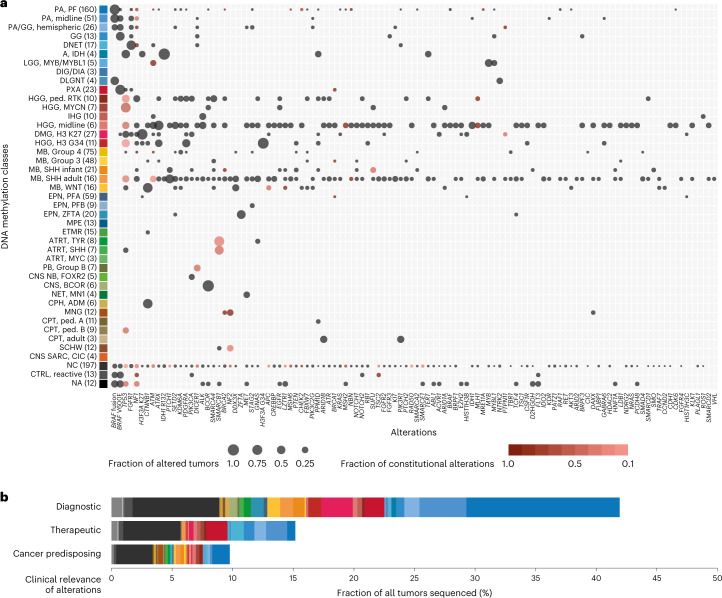
Landscape and relevance of somatic and constitutional alterations. **a**, Frequency of alterations detected in tumors (indicated by circle size) and fraction of alterations detected in corresponding constitutional DNA (color scale) across DNA methylation classes. Gene alterations (*x-*axis) are ranked by the number of affected samples. Numbers in brackets indicate tumors with available sequencing data. Only DNA methylation classes with available sequencing data for ≥3 cases and only alterations detected in ≥2 tumors are displayed. **b**, Fraction and clinical relevance of alterations detected by NGS. Colors indicate DNA methylation class. See Supplementary Table 6 for underlying data.

**Extended Data Fig. 7:**
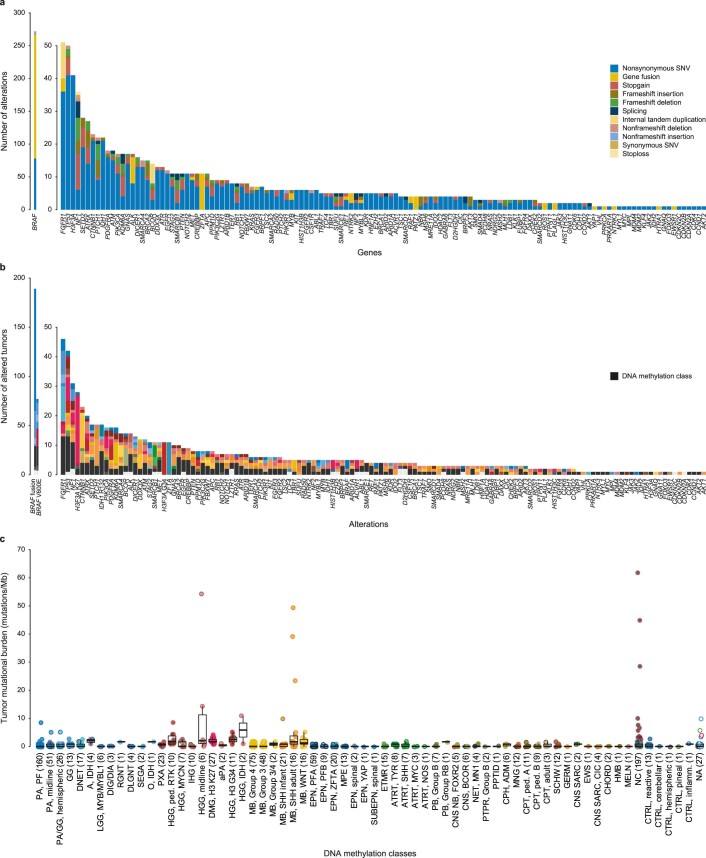
Overview of somatic alterations. **a**, Number of detected somatic alterations per gene colored by alteration type as indicated. **b**, Number of altered tumors per gene colored by DNA methylation class. **c**, Tumor mutational burden (combined scattered and boxplots) per individual tumor grouped by DNA methylation class. Each tumor is represented by a circle indicating assigned WHO-based tumor type (outline) and DNA methylation class (fill). Numbers in brackets indicate tumors with available sequencing data. Colors in (**b)** and (**c**) correspond to tumor classes as indicated in Fig. 1 and Extended Data Fig. 1. Center line, median; box limits, upper and lower quartiles; whiskers, 1.5 x interquartile range. See Supplementary Table 6 for underlying data.

Using a customized enrichment/hybrid-capture-based NGS gene panel comprising 130 genes of interest (Supplementary Table 5)^3^, complemented by RNA sequencing in selected cases^14^, we detected genetic alterations in 625 of 1,034 tumors (60.4%) (Fig. 4, Extended Data Fig. 7, Supplementary Fig. 9 and Supplementary Table 6). For the most commonly affected gene *BRAF* (272/1,034), fusion events were observed in 158 of 237 DNA methylation-defined infratentorial (124/160), midline (28/51) or cortical LGG (6/26), whereas V600E mutations were further observed in GG (7/13) and PXA (17/23). Other genes mutated in ≥2% of all tumors were *TP53* (5.1%), *FGFR1* (4.4%), *NF1* (4.2%), *H3F3A* (3.7%) and *CTNNB1* (2.2%). Recurrent alterations occurring in ≥75% of tumors (with ≥2 sequenced) in specific DNA methylation classes included histone 3 K27M in DMG, K27 (27/27), *H3F3A* G34R/V in HGG, G34 (11/11), *IDH1* in gliomas, IDH-mutant (7/7), *BCOR* ITD in CNS, BCOR (6/6), *SMARCB1* in ATRT, TYR (6/8), *DICER1* in primary intracranial DICER1-mutant sarcomas (2/2), *NF2* in spinal EPN (2/2) and *TSC1* in SEGA (2/2). A fraction of tumors unclassifiable or assigned to a control class by RF-based DNA methylation class prediction harbored diagnostically indicative alterations affecting *BRAF* (V600E, 25/214; *KIAA1549*:*BRAF*, 22/214), *IDH1* (8/214) or *H3F3A* (K27M, 2/214) as well as less clearly pathognomonic mutations. Overall, alterations considered of diagnostic relevance were detected in 41.9% of tumors (*BRAF*, 26.5%; *H3F3A*, 3.9%; *ATRX*, 2.1%; *CTNNB1*, 1.8%; *IDH1*, 1.6%; *PTCH1*, 1.5%; *ZFTA*, 1.1%; *SMARCB1*, 1.1%; and others, <1%). Alterations were considered to have therapeutic implications in 15.2% of tumors, with directly targetable alterations in *BRAF* (V600E, 7.4%), *FGFR1*/*3* (4.0%), *ALK* (0.8%), *NTRK2*/*3* (0.4%), *MET* (0.1%) and *RET* (0.1%) (Fig. 4b). Tumors considered hypermutated (with ≥10 somatic mutations per megabase (Mb)) (11/1,034, 1.1%) were among DNA methylation classes MB, SHH (4/37), HGG, midline (2/6), IDH (1/2) and unclassifiable (4/197) tumors (Extended Data Fig. 7b), with constitutional pathogenic alterations in mismatch repair (MMR)-associated genes detected in three patients with hypermutated tumors (see below). A mutational burden >5 per Mb was observed in tumors from seven of 11 patients with constitutional pathogenic alterations in MMR-associated genes.

#### Prevalence of cancer predisposition syndromes

**Extended Data Fig. 8:**
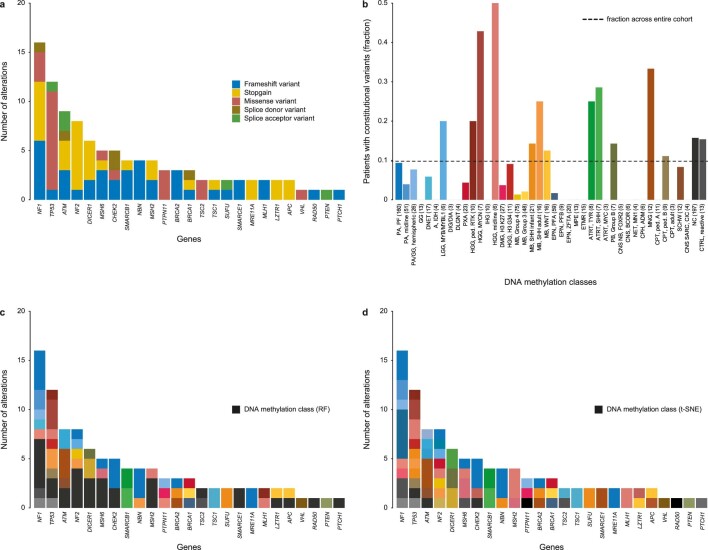
Overview of constitutional alterations. **a**, Number of detected pathogenic constitutional alterations per gene colored by alteration type as indicated. **b**, Relative fraction of patients with pathogenic constitutional variants per DNA methylation class. Numbers in brackets indicate tumors with available sequencing data. Only DNA methylation classes with available sequencing data for ≥ 3 cases are displayed. The dashed line indicates the fraction of patients with constitutional pathogenic variants across the entire cohort (at 0.98). **c** and **d**, Number of pathogenic constitutional variants per gene colored by random forest (RF)-based DNA methylation class prediction (**c**) and *t*-SNE-based DNA methylation class assignment (**d**). Colors in (**b–d**) correspond to tumor classes as indicated in Fig. 1 and Extended Data Fig. 1. See Supplementary Table 6 for underlying data.

Gene panel sequencing of leukocyte-derived DNA enabled screening for constitutional variants considered (likely) pathogenic (LPV/PV) in 1,034 patients. Cancer predisposing variants were detected in 101 of 1,034 individuals (9.8%) (Fig. 4b) affecting 25 genes (Fig. 4a, Extended Data Fig. 8, Supplementary Fig. 9 and Supplementary Table 6). The most common cancer predisposition syndromes (CPSs) were neurofibromatosis type 1 (caused by constitutional LPV/PV in *NF1*; 1.5%), Li–Fraumeni syndrome (*TP53*; 1.2%), constitutional MMR deficiency or Lynch syndrome (*MLH1*, *MSH2* and *MSH6*, 1.1%; *PMS2* was not included in the gene panel at the time of analysis), ataxia–telangiectasia and *ATM* heterozygous carriers (*ATM*, 0.9%), neurofibromatosis type 2 (*NF2*, 0.8%), DICER1 syndrome (*DICER1*, 0.6%) and rhabdoid tumor predisposition syndrome 1 (*SMARCB1*, 0.4%). LPV/PV in other genes occurred at lower frequencies (<0.5%). Known associations included *NF1* in LGG and *SMARCB1* in atypical teratoid/rhabdoid tumor (AT/RT) (Supplementary Fig. 9a,e). Additional findings included constitutional *TP53* variants enriched in MYCN-activated HGG; *MLH1*, *MSH2* and *MSH6* in RTK-activated and midline HGG classes (Extended Data Fig. 8 and Supplementary Fig. 9b); and notable findings including a previously unidentified *PTPN11* variant in a patient with an H3 K27-altered DMG. We also observed a substantial proportion of patients with pathogenic constitutional alterations whose tumors were not readily classifiable by RF-based DNA methylation class prediction (31/101, 30.7%), of which most displayed high-grade (13/31, 41.9%) or low-grade (4/31, 12.9%) glioma histology, in line with *t*-SNE-based DNA methylation class assignment (15/31, 48.4%), including three *IDH1*-mutant astrocytomas. Indications for cancer predisposition were documented at national study headquarters in only 37 of 101 (36.6%) patients in whom we detected constitutional pathogenic variants, indicating a high proportion of previously unknown CPS among affected individuals and their families. Due to the lack of routine copy number assessment in constitutional patient DNA, constitutional copy number variations of *SMARCB1* were not reported in two patients with AT/RT and a known rhabdoid tumor predisposition syndrome where data were suggestive of a heterozygous deletion.

### Interdisciplinary tumor board discussions

**Extended Data Fig. 9:**
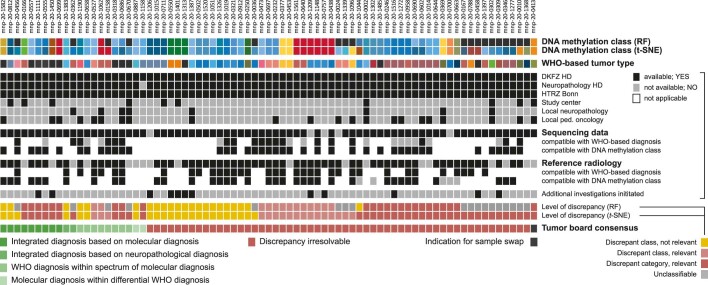
Tumor board discussions of cases with discrepant classification. Details of tumor board discussions of tumors with discrepant classification by random forest (RF)-based and *t*-SNE-based DNA methylation class assignment (as in Fig. 3c and Supplementary Fig. 8; upper two rows) and WHO-based tumor type (third row). Colors in rows 1–3 correspond to tumor types and classes as indicated in Fig. 1 and Extended Data Fig. 1. Tumor board participants and availability of additional information (gene panel sequencing, reference radiology) is indicated by black boxes as well as compatibility with DNA methylation- and WHO-based tumor classification. Levels of discrepancy (corresponding to Fig. 3c and Supplementary Fig. 8) and tumor board consensus are categorized in bottom rows. See Supplementary Table 1 for underlying data.

Cases with discrepant neuropathological WHO-based and DNA methylation-based classification were discussed in a weekly interdisciplinary tumor board (Extended Data Fig. 9 and Supplementary Table 1). Focusing on discrepancies after DNA methylation class assignment by *t*-SNE inspection, 70.1% of discussed discrepancies were considered clinically relevant. Additional gene panel sequencing data and reference neuroradiological evaluation were available in 93.5% and 76.6% of cases, respectively, and considered compatible with both WHO-based (63% and 100%) and DNA methylation-based (100% and 85%) classification in most cases. Variants detected by NGS considered inconsistent with WHO tumor type predominantly occurred as *BRAF* or *MYBL1* alterations in HGG defined by WHO criteria (8/14). Additional investigations (such as targeted sequencing or FISH) were initiated in 15.6%. Constellations enabled a consensus in 27.3% of discussed cases, in which an integrated diagnosis was based on DNA methylation class (42.9%) or WHO tumor type (9.5%); the WHO tumor type was within the histopathological spectrum of the DNA methylation class (38.1%); or the DNA methylation class was considered as a differential diagnosis by reference neuropathological evaluation (9.5%). Discrepancies remained irresolvable in most discussed cases (71.4%). Review of WHO-defined anaplastic astrocytomas and glioblastomas displaying DNA methylation profiles of lower-grade gliomas (frequently occurring in infants and young children) indicated increased mitotic activity, in particular with aberrant (atypical) mitotic figures, as the main reason for assigning a high grade, with thrombosed vessels or palisading necrosis as criteria for anaplasia in individual cases. One sample swap (<0.1%) occurred during molecular analysis and was detected upon discussion.

### Risk stratification for patients with HGG

**Fig. 5:**
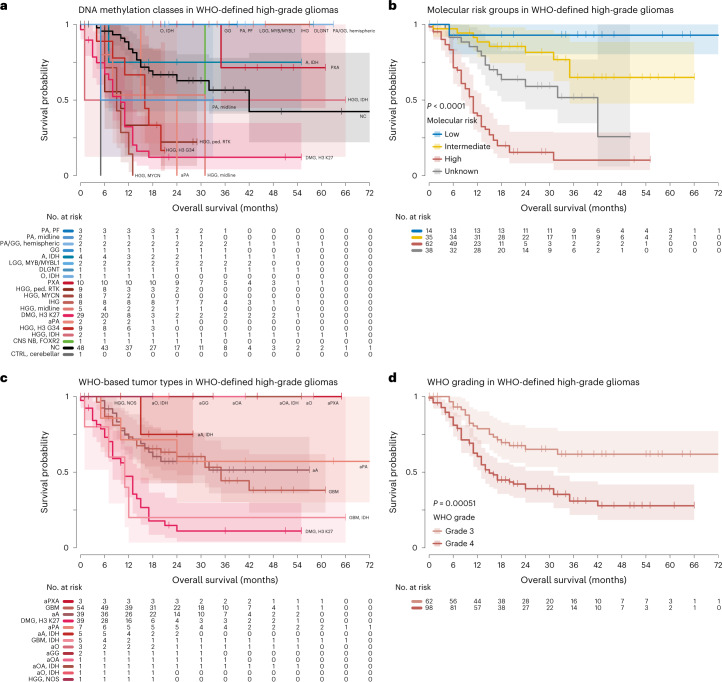
Molecular risk stratification of pediatric patients with HGG. Kaplan–Meier estimates of OS in patients with WHO-defined HGGs according to DNA methylation class (**a**), molecular risk group (**b**), WHO-based tumor type (**c**) and WHO grade (**d**). Colors in **a** and **c** correspond to tumor types and classes as indicated in Fig. 1 and Extended Data Fig. 1. Shaded areas indicate the 95% confidence interval for each Kaplan–Meier estimate (solid lines). See Supplementary Table 1 for underlying and further data.

Given the recurring constellation of HGG according to WHO criteria with DNA methylation profiles of lower-grade gliomas (Fig. 2), we stratified patients with WHO-defined HGG into molecular risk groups. Data on survival and treatment modalities were available for 952 enrolled patients (79.1%; Supplementary Table 1), including 162 patients with WHO-defined HGG. Median follow-up was 22 months (range 0–192 months) after diagnosis. Tumors from high-risk DNA methylation classes (DMG, K27M; HGG, G34; HGG, midline; HGG, MYCN; HGG, RTK) were associated with poor overall survival (OS), whereas HGG from intermediate-risk (A, IDH; HGG, IDH; aPA; PXA; IHG; CNS NB, FOXR2) and low-risk (PA, PF; PA, midline; PA/GG, hemispheric; GG; LGG, MYB/MYBL1; DLGNT) DNA methylation classes were associated with significantly longer OS (*P* < 0.001, log-rank test) (Fig. 5a,b). Patients in the low-risk group included four children in complete remission (two of them 34 months and 41 months after tumor resection and following a watch-and-wait strategy) and only five patients who had received both radiotherapy and chemotherapy (Supplementary Table 1). Similar results in this group were obtained when using DNA methylation class assignment by *t*-SNE analysis (Supplementary Fig. 10) or defining the HGG cohort for analysis by DNA methylation classes (Supplementary Fig. 11a,b). There was also a significant, yet less discriminatory, difference when comparing tumors assigned WHO grade 3 with WHO grade 4 (*P* = 0.0051) (Fig. 5c,d), and WHO grades 1–2 (PXA, WHO grade 2 in 9/13 cases) indicated improved OS among DNA methylation-defined HGG (Supplementary Fig. 11c,d). Additional survival analyses by WHO-based tumor type and DNA methylation class in LGG (Supplementary Fig. 12), MB (Supplementary Fig. 13), EPN (Supplementary Fig. 14) and EMB/PIN (Supplementary Fig. 15) indicated differences largely known from previous retrospective studies.

### Advancement of automated DNA methylation class prediction

**Extended Data Fig. 10:**
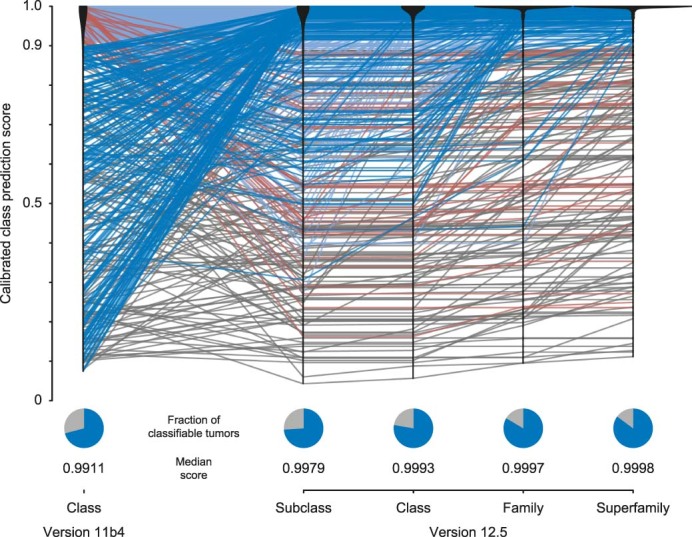
Advancement of automated DNA methylation class prediction. Calibrated class prediction scores of random forest-based DNA methylation class prediction in version 11b4 and prediction scores for DNA methylation levels: subclasses, classes, class families, and superfamilies in version 12.5 (upper panel). Every line represents one tumor; light blue: classifiable by both versions; dark blue: classifiable by version 12.5 only; red: classifiable by version 11b4 only; grey: non-classifiable by both versions. Black violin plots represent density estimates for each version and level. Pie charts (lower panel) indicate the fractions of classifiable tumors (calibrated scores ≥ 0.9, blue) and unclassifiable tumors (calibrated scores < 0.9, grey) by each version and level. See Supplementary Table 1 for underlying data.

To evaluate the advancement of RF-based DNA methylation class prediction, we applied version 11b4 (publicly released in October 2017)^4^ and version 12.5 (released in January 2022) of the algorithm to the DNA methylation dataset of 1,124 tumors (Extended Data Fig. 10 and Supplementary Table 1). By increasing the total class number and introducing a hierarchy of DNA methylation subclasses (184), classes (147), class families (81) and superfamilies (66), the total number of tumors that could not readily be assigned to any tumor category decreased from 29% in version 11b4 to 15% in version 12.5. At the same time, 32 tumors (2.9%) that were assigned to a distinct class in version 11b4 did not reach the threshold score of 0.9 for any class or family in version 12.5. Another 135 tumors (12.0%, 126 of which were deemed classifiable by *t*-SNE analysis) remained unclassifiable in both versions of the RF-based algorithm. In 58 of 167 samples unclassifiable by version 12.5, genetic alterations indicative of a DNA methylation class were detected by NGS in *BRAF* (42/167), *IDH1* (5/167), histone 3 genes (4/167), *CTNNB1* (3/167), *ALK* (2/167), *SMARCB1* (1/167) and *YAP1* (1/167).

## Discussion

In contrast to the unbiased approach presented here, previous studies applying similar techniques were largely performed in retrospect^4,15^, aiming specifically to subgroup archived cohorts defined by WHO tumor types^16–19^ or to characterize novel CNS tumor groups based on distinct DNA methylation patterns^12,13,20–23^, and smaller-scale prospective studies focused explicitly on tumors challenging to classify by conventional neuropathology and/or did not follow-up on patient outcome^5–7^.

Our data support the incorporation of DNA methylation-based classification as included in the 5th edition of the WHO classification of CNS tumors as a desirable diagnostic criterion for many tumor types and an essential criterion for some otherwise difficult to diagnose^2,24^. Adding a DNA methylation (sub)class further refines the molecular layer of a coherent integrated diagnosis in most cases, which is becoming increasingly important in the era of molecularly informed patient stratification and subgroup-specific therapies. DNA methylation analysis has the potential to increase certainty in tumors with a suspected diagnosis and to establish a valid diagnosis in some samples where no neoplastic cells can be detected by neuropathological examination alone. On the other hand, contamination by non-neoplastic cells can be a limitation for reaching the diagnostic threshold for DNA methylation-based CNS tumor class prediction and underlines the importance of thorough neuropathological assessment^25^.

The enrichment of discrepant classifications in gliomas suggests that this group of pediatric patients may currently benefit most from integrating DNA methylation analysis in standard neuropathological practice. A substantial fraction of histologically defined HGGs present with DNA methylation profiles resembling those of lower-grade lesions. Our interdisciplinary tumor board discussions show that—especially in the absence of pathognomonic mutations or fusions—a diagnostic gold standard is usually missing, making consensus on an integrated diagnosis often difficult to reach. In the ongoing debate concerning the clinical behavior of these tumors, our follow-up data indicate improved outcome, similar to patients with histologically defined LGG. Using prospectively assigned DNA methylation classes to stratify patients with HGG into molecular risk groups predicted prognosis more accurately than WHO grading and should be considered for clinical decision-making in such constellations. Some of these are already incorporated in the current WHO classification, exemplified by exclusion of anaplasia as an essential diagnostic criterion for *MYB*-altered or *MYBL1*-altered diffuse astrocytomas^2^. Increased mitotic activity as the main reason for diagnosing HGGs in infants and young children whose tumors display DNA methylation patterns of lower-grade gliomas warrants future studies to better define cutoffs for tumor mitotic activity in this age group. The DNA methylation class comprising both WHO grades 2 and 3 of PXA (based on mitotic count^2^, here provisionally categorized as HGG) was associated with an intermediate prognosis compared to both HGG and LGG within our follow-up period, rendering grading for this class difficult and re-visiting these data in the future necessary.

For tumors not readily classifiable by RF-based class prediction, subjecting DNA methylation data to advanced analyses such as *t*-SNE alongside suitable reference cohorts can be instrumental in determining tumor type. Tumors with class prediction scores slightly below the threshold of 0.9 are typically projected onto or in close proximity to reference tumors of a DNA methylation class and may still be reliably assigned to that class (Supplementary Fig. 16)^25^. In contrast, tumors with overall low scores are often projected in between reference tumor classes. They may indicate the existence of yet unknown DNA methylation classes, especially when clustering together with other difficult-to-classify samples over time. Results from our study fed into a constantly growing database of more than 100,000 tumors that allows for identifying such clusters, exploring their associated molecular, pathological and clinical features, and iteratively introducing them as new reference DNA methylation (sub)classes into the RF-based class prediction algorithm^12,13,20–22,26^, resulting in lower rates of unclassifiable tumors applying in its latest version. The requirement of careful visual inspection and (subjective) interpretation of output generated by *t*-SNE analyses, however, remain a caveat when used for clinical decision-making.

The associations between certain copy number alterations and DNA methylation classes in our current cohort confirm the benefit of integrating DNA copy number alterations derived from DNA methylation arrays into diagnostic considerations^25^. At the time of primary diagnosis, DNA methylation-based CNS tumor classification and copy number profiling is ideally complemented by targeted NGS of a neuro-oncology-specific gene panel (or equivalent approaches) designed to detect diagnostically and/or therapeutically relevant alterations from tumor and constitutional DNA^3^. The presence of a pathognomonic alteration (for example, in *BRAF*, histone 3 variants, *IDH*, *ZFTA*, *BCOR*, *MN1* and others) corroborates a specific diagnosis in tumors with discrepant classification or inconclusive DNA methylation analysis. As molecularly informed treatment strategies are becoming increasingly feasible as first-line options, identifying a tumor’s mutational makeup, including directly targetable alterations, will be essential in guiding patients toward optimal treatment, as demonstrated by targeting BRAF V600E, FGFR, ALK and NTRK in (among others) pediatric gliomas^27–32^. In selected tumors, subsequent RNA sequencing from the same FFPE sample (as performed here) represents a feasible approach to detect fusions with immediate impact on patient care^14,23^.

Our results suggest previous assessments of pathogenic constitutional variants underlying CNS tumor development (in approximately 10% of patients) to appear broadly robust^15^ and an enrichment of Li–Fraumeni syndrome, Lynch syndrome and constitutional MMR deficiency underlying H3 wild-type HGG. We, therefore, recommend genetic counseling and testing for pediatric patients with H3 wild-type HGG (in addition to existing guidelines^33,34^). The clinical information retrieved through national study headquarters indicates that most patients were not known or suspected to carry pathogenic constitutional variants, similarly to previous observations beyond patients with CNS tumors^35,36^. This highlights the importance of diligent consultation of patients and their families, considering that more than 95% of study participants and parents elected to be informed about constitutional pathogenic variants detected by NGS. Detection of CPSs at primary diagnosis brings added advantages over precision oncology programs designed for relapsed or progressive malignancies^35^ by enabling appropriate adaptation of treatment approaches already in the frontline setting—for example, avoiding ionizing irradiation to reduce the risk of secondary tumors in patients with Li–Fraumeni syndrome^37^ or considering upfront immune checkpoint inhibition in children with constitutional DNA replication repair deficiency^38,39^. The high fraction of tumors not readily classifiable by RF-based class prediction in patients with CPSs may be addressed by augmenting future reference cohorts with syndrome-associated tumors^40^.

Although we consider the median turnaround time of ~21 days for the centralized generation and interpretation of DNA methylation profiling and targeted NGS results acceptable, the regulatory and logistic framework of our study resulted in delays primarily affecting pre-analytical steps performed at the level of more than 60 local centers, posing a challenge especially for hospitals with lower patient recruitment. DNA methylation analysis has recently been decentralized and is now being performed at more than five experienced neuropathology centers across Germany as part of their immediate reference evaluation, minimizing total turnaround times between operation and reporting down to less than 28 days. Although targeted tumor/blood NGS is currently being performed in a similar timeframe, it cannot be initiated without informed consent from patients/parents indicating their desire to (not) be informed about potential relevant constitutional alterations. Together with the need to obtain and ship a patient blood sample, this may cause pre-analytical delay if not initiated early.

Providing multi-omic data from as few as ten unstained sections of FFPE tissue, our study produced a high level of information at reasonable costs and with a very low dropout rate of ~5% of tumors. The benefits of our program and their impact on clinical patient management have prompted German national health insurance companies to cover the expenses for DNA methylation analysis and gene panel sequencing (from both tumor tissue and blood leukocytes) as part of the reference services of the nationwide multi-disciplinary ‘Treatment Network HIT’ for children and adolescents with newly diagnosed CNS tumors. This sets an excellent example of direct and rapid translation of scientific innovation into routine clinical practice, substantially improves the standard of care in German pediatric neuro-oncology and may serve as a blueprint for other countries.

## Methods

### Patient population, samples and clinical data collection

Patients were recruited between April 2015 and March 2019 from childhood cancer centers cooperating within the German Society for Pediatric Oncology/Hematology (GPOH), the Swiss Paediatric Oncology Group (SPOG) and the Australian & New Zealand Children’s Haematology/Oncology Group (ANZCHOG) in accordance with ethics board approval from the ethics committee of the Medical Faculty Heidelberg as well as local institutes. Patient sex and/or gender were not considered in the design of the study. Inclusion criteria comprised age ≤21 years at primary diagnosis of a CNS neoplasm and availability of FFPE tumor tissue. FFPE tumor tissue for reference neuropathological assessment and patient blood samples were collected at the Brain Tumor Reference Center (HTRZ) of the German Society for Neuropathology and Neuroanatomy (DGNN; Department of Neuropathology, Bonn, Germany). FFPE tumor tissue and patient blood samples were forwarded to the Clinical Cooperation Unit Neuropathology at the German Cancer Research Center (DKFZ) for molecular analyses in accordance with research ethics board approval of the University of Heidelberg. Clinical patient data were collected at the DKFZ through national study headquarters of the German HIT network of the GPOH, SPOG and ANZCHOG, using standardized case report forms within the framework of clinical trials. Evidence or clinical signs of cancer predisposition were reported to national study headquarters by local participating centers as part of those case report forms but not reviewed. Additional clinical data from 84 patients with WHO-defined HGG were obtained by reviewing primary records provided by local treating centers. Patient sex was determined by physical examination by the treating physician responsible for patient registration. No disaggregated information on patient sex and gender was collected in this study.

### Informed consent

The MNP 2.0 study complies with the principles of the Declaration of Helsinki in its current version. Informed consent from adult patients or parental consent was obtained for all patients before enrollment. As part of consenting, patients or parents decided if they wanted to be informed about constitutional variants indicative of a CPS (890/935, 95.2%) or not (45/935, 4.8%). In cases for which this decision was not forwarded upon registration (269/1,204, 22.3%) and sequencing data were available (157/1,034, 15.2%), information on constitutional variants was not reported to treating physicians, but pseudonymized data were included in further aggregated analyses presented here, as part of the approved protocol. Only constitutional variants considered pathogenic or likely pathogenic were reported (see below).

### CNS tumor nomenclature

To conform with the 2021 WHO Classification of Tumors of the CNS, the term ‘type’ is used for specific diagnoses recognized by the WHO (termed ‘entity’ in previous editions; for example, ‘pilocytic astrocytoma’), and the term ‘subtype’ is used for subgroups thereof (termed ‘variant’ in previous editions)^2,24^. Multiple CNS tumor types are grouped into ‘categories’ (for example, ‘low-grade glioma’). To conform with the 2021 WHO Classification of Tumors of the CNS, WHO tumor grades are expressed in Arabic numerals even though based on previous editions^1,41^. For DNA methylation-based classification, the term ‘class’ refers to a distinct DNA methylation class^4^ (for example, ‘pilocytic astrocytoma, posterior fossa’), and multiple classes are grouped into ‘categories’ corresponding to the category level of WHO-based tumor types. A hierarchy of ‘subclasses’, ‘classes’, ‘class families’ and ‘superfamilies’ was introduced in version 12.5 of the DNA methylation-based CNS tumor classification algorithm.

### Color coding

Palettes of optimally distinct colors for CNS tumor categories and types/classes (as depicted in Extended Data Fig. 1) were generated and refined using *I want hue* developed by Mathieu Jacomy at the Sciences-Po Medialab (http://medialab.github.io/iwanthue) and *Graphical User Interface to Pick Colors in HCL Space* by Claus O. Wilke, Reto Stauffer and Achim Zeileis (http://hclwizard.org:3000/hclcolorpicker). Corresponding DNA methylation classes and WHO-based diagnoses share the same color hue; overlapping DNA methylation classes and WHO-based diagnoses share shades of the same color hue (that is, different luminance). DNA methylation classes and WHO-based diagnoses from the same tumor category share a similar color hue spectrum.

### Reference neuropathological evaluation

Central reference neuropathological evaluation was performed at the HTRZ (Department of Neuropathology, Bonn, Germany) according to the criteria defined by the respective applicable version of the WHO classification at the time of diagnosis—that is, 4th (2015–2016) and revised 4th (2016–2019) editions^1,41^. Diagnostic workup included conventional stainings such as hematoxilin & eosin staining and silver impreganation, immunohistochemical analysis of differentiation, cell lineage and proliferation markers and for mutant proteins as well as molecular pathological assays where appropriate for reaching a WHO-conform diagnosis. Tumor tissue from 21 of 707 patients (3.0%; recorded until 15 February 2018) was sufficient only for reference neuropathological assessment.

### Molecular genetic analyses

Per protocol, ten unstained sections of FFPE tissue were requested for molecular genetic analyses. In 980 of 1,161 cases with detailed documentation (84%), a complete set of one HE-stained section, three sections at 4 µm and ten sections at 10 µm or an FFPE tissue block were available (Supplementary Table 1). In 1,093 of 1,161 cases (94%), a minimum of ten sections at 10 µm were available. Testing also proceeded if fewer than ten sections at 10 µm (range: 2–9 sections; median: six sections) were available (59/1,161, 5%). In 11 of 1,161 cases (1%), DNA extracted at the stage of reference neuropathological evaluation was provided. Although aiming to extract DNA from tissue areas with more than 70% tumor cell content, this was not a prerequisite for molecular genetic analyses.

Nucleic acid extraction, DNA methylation and copy number analysis using the Infinium HumanMethylation450 (*n* = 187) and MethylationEPIC (*n* = 937) BeadChip arrays (Illumina) and tumor/constitutional DNA sequencing using a customized enrichment/hybrid-capture-based NGS gene panel were performed at the Department of Neuropathology, Heidelberg University Hospital, as previously described^3,4^. The NGS panel comprised the entire coding (all exons ±25 bp) and selected intronic and promoter regions of 130 genes (Supplementary Table 5) and was designed to detect single-nucleotide variants (SNVs), small insertions/deletions (InDels), exonic re-arrangements and recurrent fusion events. For selected samples (*n* = 41), RNA sequencing was performed as previously described^14^. Selection criteria for RNA sequencing included indications for fusion events inferred by targeted DNA sequencing or copy number data derived from DNA methylation arrays, assignment to DNA methylation classes known to be associated with fusion events (such as infantile hemispheric gliomas or *MYB*/*MYBL1*-altered LGGs) and unclassifiable tumors in which RNA sequencing was deemed potentially informative.

NGS data were processed and analyzed as previously described^3,14^. In addition to automated SNV and InDel calling, hotspots in *BRAF*, *H3F3A*, *IDH1*, *BCOR* and *FGFR1* were manually screened for alterations using the Integrative Genomics Viewer (IGV)^42^. Tumor mutational burden was calculated as the total number of somatic SNVs and InDels per Mb of investigated genomic sequence (including synonymous SNV and hotspot mutations). NGS data were not analyzed for copy number variations. Relevant constitutional alterations identified by NGS of leukocyte-derived DNA were technically validated by Sanger sequencing at the Institute of Human Genetics at Heidelberg University Hospital. Constitutional alterations in a predefined list of 47 known cancer predisposition genes included in the gene panel (Supplementary Table 7) were assessed by human geneticists according to American College of Medical Genetics and Genomics (ACMG) criteria^43^, and only likely pathogenic (ACMG class 4) or pathogenic (ACMG class 5) variants were reported to the treating physician, and genetic counseling of the patient and the family was recommended.

DNA methylation-based classification of tumor samples was performed using an RF classifying algorithm as published previously^4^, using, in each case, the latest applicable CNS tumor classifier version at the time of diagnosis—that is, version 9.0 (2015; *n* = 64), version 11.0 (2015–2016; *n* = 95), version 11b2 (2016–2017; *n* = 325), version 11b4 (2017–2019; *n* = 658) and version 12.5 (applied for aggregated re-analysis of all 1,124 tumors as depicted in Extended Data Fig. 10) (https://www.molecularneuropathology.org/mnp/). In version 9.0, a tumor was assigned to a DNA methylation class if its raw RF-based class prediction score was within the interquartile range of class prediction scores of the respective reference class. After the introduction of score calibration (version 11.0), a DNA methylation class was assigned to a sample when its calibrated class prediction score reached the threshold of ≥0.9 for a reference class^4^. *t*-SNE analysis of DNA methylation data from the study cohort was performed alongside 89 published reference DNA methylation classes^4^ after removal of five duplicate samples from the reference cohort. DNA methylation data from 208 of 1,124 samples in this study cohort were part of the reference cohort used to train version version 12.5 of the RF classifying algorithm.

Discrepancies between WHO tumor type and DNA methylation class were considered clinically relevant if the diagnosis according to DNA methylation-based classification would have affected clinical patient management by changing the recommended treatment protocol and, therefore, (1) applying or omitting chemotherapy, (2) applying or omitting radiotherapy or (3) applying a different chemotherapy regimen. Recommendations for clinical patient management were based on phase 3 clinical trial protocols endorsed by the brain tumor ‘Treatment Network HIT’ of the GPOH between 2015 and 2019.

Cancer cell fraction and tumor purity were predicted in silico from DNA methylation data by deconvolution of tumor composition (MethylCIBERSORT)^44^ and RF-based tumor purity prediction (RF_Purify)^45^, respectively (Supplementary Fig. 17). There was a direct correlation between the two methods (Pearson correlation: 0.86), but neither of the two estimates for tumor cell content correlated with RF class prediction scores (using version 11b4 across the entire cohort). Lower tumor cell content was predominantly observed in LGG but did not seem to necessarily impair class prediction. Overlaying estimated tumor cell content with *t*-SNE analyses showed a clear tendency for tumors with lower tumor cell content to cluster together and in close proximity of the non-neoplastic reference DNA methylation class ‘Control tissue, reactive tumor microenvironment’.

Enhanced copy number variation analysis using Illumina DNA methylation arrays was performed using the R package conumee^46^. DNA copy number state of the genomic locus containing *CDKN2A/B* in *BRAF* V600E-positive and *BRAF* fusion-positive tumors was assessed by visual inspection of resulting segmented copy number data using IGV^42^. Summary copy number plots to display rates of copy number gains and losses per DNA methylation class with a minimum sample size of five were generated using an in-house R script (https://github.com/dstichel/CNsummaryplots). GISTIC2.0 (version 2.0.23) analyses were performed to identify genes targeted by somatic copy number variations per DNA methylation class with a minimum sample size of five via the online platform GenePattern (https://www.genepattern.org/) using default settings^47^. All other computational analyses were performed using the programming language R (ref. ^48^).

### Sample processing timelines

Total processing time from operation to reporting of molecular results ranged from 30 days to 290 days (median 77 days, excluding 79 patients registered >100 days after operation) (Supplementary Fig. 3a). Most time was consumed for patient registration (median 14 days; range 0–95 days) and data generation (median 18 days; range 5–59 days, with DNA methylation analyses completed before patient registration as part of local neuropathological diagnostics in seven cases). There was no considerable change in sample processing times throughout the recruitment period, but there was a trend toward earlier patient registration in centers with higher recruitment (Supplementary Fig. 3b,c).

### Interdisciplinary tumor board discussion

Interdisciplinary tumor board discussions of cases with divergent reference neuropathological and molecular classification were held with a maximum of four cases per week. Discussions included participants from the DKFZ (Division of Pediatric Neurooncology), Heidelberg University Hospital (Department of Neuropathology), the Brain Tumor Reference Center (Bonn, Germany) and the Neuroradiology Reference Center (Würzburg/Augsburg, Germany). Participation of local pediatric oncologists and neuropathologists and representatives of the GPOH/SPOG/ANZCHOG study centers was encouraged but optional.

In cases with discrepant findings, results of DNA methylation analysis and gene panel sequencing were initially forwarded only to treating physicians after interdisciplinary tumor board discussion and included a summary of the tumor board consensus. In April 2016, the study protocol was amended, and molecular results were provided immediately with a caveat that the report was considered preliminary until tumor board discussion; a final report including the tumor board consensus was issued thereafter.

### Risk stratification of patients with HGG

Patients with HGGs (WHO grade 3–4) diagnosed by reference neuropathological evaluation according to the criteria of the WHO classification of tumors of the CNS were assigned to molecular risk groups based on the following molecular criteria. High risk: DNA methylation classes of HGG, G34; DMG, K27; HGG, MYCN; HGG, midline; HGG, RTK; in tumors unclassifiable by RF-based DNA methylation class prediction or without DNA methylation data: presence of an H3 K27M (*n* = 1) or H3 G34R/V (*n* = 1) mutation. Intermediate risk: DNA methylation classes of A, IDH; HGG, IDH; O, IDH; aPA; PXA; IHG; CNS NB, FOXR2; in tumors unclassifiable by RF-based DNA methylation class prediction: presence of an *IDH1/2* R132H mutation (*n* = 7); presence of a fusion involving *ALK* (*n* = 4), *NTRK* (*n* = 2), *ROS1* (*n* = 1) or *MET* (*n* = 1); co-occurrence of *BRAF* V600E mutation and *CDKN2A/B* homozygous deletion (*n* = 2). Low risk: DNA methylation classes of PA, PF; PA, midline; PA/GG, hemispheric; LGG, MYB/MYBL1; GG; DLGNT; in tumors with low tumor cell content unclassifiable by RF-based DNA methylation class prediction or without DNA methylation data: presence of a *BRAF* fusion (*n* = 16); presence of a *BRAF* V600E mutation in absence of a *CDKN2A/B* deletion (*n* = 24). Unknown risk: DNA methylation class of non-neoplastic control tissue or pattern unclassifiable in absence of abovementioned alterations. Not assessed: DNA methylation analysis not performed, targeted gene panel sequencing not performed or without detection of abovementioned alterations. By *t*-SNE-based DNA methylation class assignment, molecular high-risk HGG additionally included HGG of the posterior fossa. Intermediate-risk HGG additionally included DGONC^20^. Low-risk HGG additionally included LGG, not otherwise specified (NOS). Tumors with *t*-SNE-based assignment to novel DNA methylation classes with unknown clinical behavior, such as tumors with *PATZ1* fusions^12^ or *PLAGL1* fusions^13^, were excluded.

### Statistical analysis of molecular and clinical data

Correlation between classification into individual WHO-based tumor types and DNA methylation-based tumor classes was tested by calculating the phi coefficient between a sample × WHO type and a sample × DNA methylation class matrix. The distribution of discrepant constellations between WHO-based tumor type and DNA methylation class among tumor categories was tested using a Fisher’s exact test. Kaplan–Meier analysis was performed to estimate the survival time of patients from different CNS tumor groups, and a log-rank test was performed to compare survival distributions between independent groups. Pairwise comparisons between groups were corrected for multiple testing using the Benjamini–Hochberg method. OS was defined as time from date of initial diagnosis until death of any cause. Surviving patients were censored at the date of last follow-up. Event-free survival was calculated from date of diagnosis until event, defined as relapse after complete resection, clinical or radiological progression, start of non-surgical/adjuvant therapy or death of any cause. Patients without event were censored at the date of last follow-up. Data visualization and statistical analyses were performed using the programming language R (ref. ^48^). Tumor location was visualized for DNA methylation classes with a minimum sample size of five by adapting an R package for anatomical visualization of spatiotemporal brain data^49^.

### Reporting summary

Further information on research design is available in the Nature Portfolio Reporting Summary linked to this article.

## Online content

Any methods, additional references, Nature Portfolio reporting summaries, source data, extended data, supplementary information, acknowledgements, peer review information; details of author contributions and competing interests; and statements of data and code availability are available at 10.1038/s41591-023-02255-1.

### Supplementary information

Supplementary Information Supplementary Figs. 1–17 and Supplementary Table legends.

Reporting Summary

Supplementary Tables 1 and 3–7

Supplementary Table 2

## Data Availability

DNA methylation data generated during this study have been deposited in the National Center for Biotechnology Informationʼs Gene Expression Omnibus (http://www.ncbi.nlm.nih.gov/geo) under accession number GSE215240. DNA methylation data used as a reference^4^ have been deposited under accession number GSE90496. Targeted next-generation DNA sequencing data have been deposited at the European Genome-phenome Archive (http://www.ebi.ac.uk/ega/) under accession number EGAS00001006680. Access can be requested from the Data Access Committee and is linked to a data access agreement. All source data to replicate our results are provided within supplementary tables.

## Code availability

The R package conumee used for enhanced copy number variation analysis using Illumina DNA methylation arrays is available at Bioconductor (https://bioconductor.org/packages/release/bioc/html/conumee.html). The R script used to display rates of copy number gains and losses per DNA methylation class is available at https://github.com/dstichel/CNsummaryplots. GISTIC2.0 to identify genes targeted by somatic copy number variations per DNA methylation is accessible via GenePattern (https://www.genepattern.org/modules/docs/GISTIC_2.0). The R package cerebroViz adapted for visualization of tumor location is available at https://github.com/ethanbahl/cerebroViz. The code underlying the random forest-based algorithm for DNA methylation-based CNS tumor classification was previously described^4^ and is available at https://github.com/mwsill/mnp_training.
