# Author Correction: Multiomic neuropathology improves diagnostic accuracy in pediatric neuro-oncology

**DOI:** 10.1038/s41591-023-02652-6

**Published:** 2023-10-24

**Authors:** Dominik Sturm, David Capper, Felipe Andreiuolo, Marco Gessi, Christian Kölsche, Annekathrin Reinhardt, Philipp Sievers, Annika K. Wefers, Azadeh Ebrahimi, Abigail K. Suwala, Gerrit H. Gielen, Martin Sill, Daniel Schrimpf, Damian Stichel, Volker Hovestadt, Bjarne Daenekas, Agata Rode, Stefan Hamelmann, Christopher Previti, Natalie Jäger, Ivo Buchhalter, Mirjam Blattner-Johnson, Barbara C. Jones, Monika Warmuth-Metz, Brigitte Bison, Kerstin Grund, Christian Sutter, Steffen Hirsch, Nicola Dikow, Martin Hasselblatt, Ulrich Schüller, Arend Koch, Nicolas U. Gerber, Christine L. White, Molly K. Buntine, Kathryn Kinross, Elizabeth M. Algar, Jordan R. Hansford, Nicholas G. Gottardo, Martin U. Schuhmann, Ulrich W. Thomale, Pablo Hernáiz Driever, Astrid Gnekow, Olaf Witt, Hermann L. Müller, Gabriele Calaminus, Gudrun Fleischhack, Uwe Kordes, Martin Mynarek, Stefan Rutkowski, Michael C. Frühwald, Christof M. Kramm, Andreas von Deimling, Torsten Pietsch, Felix Sahm, Stefan M. Pfister, David. T. W. Jones

**Affiliations:** 1 Hopp Children’s Cancer Center Heidelberg (KiTZ), Heidelberg, Germany; 2 Division of Pediatric Glioma Research, German Cancer Research Center (DKFZ) and German Cancer Consortium (DKTK), Heidelberg, Germany; 3 Department of Pediatric Oncology, Hematology & Immunology, Heidelberg University Hospital, Heidelberg, Germany; 4 Department of Neuropathology, Charité - Universitätsmedizin Berlin, corporate member of Freie Universität Berlin and Humboldt-Universität zu Berlin, Berlin, Germany; 5 German Cancer Consortium (DKTK), Partner Site Berlin, German Cancer Research Center (DKFZ), Heidelberg, Germany; 6 Department of Neuropathology, DGNN Brain Tumor Reference Center, University of Bonn, Bonn, Germany; 7 Laboratory of Neuropathology, Paulo Niemeyer State Brain Institute, Rio de Janeiro, Brazil; 8 D’Or Institute for Research and Education (IDOR), Rio de Janeiro, Brazil; 9 Institute of Pathology, Heidelberg University Hospital, Heidelberg, Germany; 10 Department of Neuropathology, Heidelberg University Hospital, Heidelberg, Germany; 11 Institute of Neuropathology, University Medical Center Hamburg-Eppendorf, Hamburg, Germany; 12 Clinical Cooperation Unit Neuropathology, German Cancer Research Center (DKFZ) and German Cancer Consortium (DKTK), Heidelberg, Germany; 13 Department of Neurological Surgery, Helen Diller Research Center, University of California, San Francisco, San Francisco, CA USA; 14 Division of Pediatric Neurooncology, German Cancer Research Center (DKFZ) and German Cancer Consortium (DKTK), Heidelberg, Germany; 15 Department of Pediatric Oncology, Dana-Farber Cancer Institute, Boston, MA USA; 16 Broad Institute of MIT and Harvard, Cambridge, MA USA; 17 Omics IT and Data Management Core Facility, German Cancer Research Center (DKFZ) and German Cancer Consortium (DKTK), Heidelberg, Germany; 18 Department of Diagnostic and Interventional Neuroradiology, University Hospital of Würzburg, Würzburg, Germany; 19 Neuroradiological Reference Center for the Pediatric Brain Tumor (HIT) Studies of the German Society of Pediatric Oncology and Hematology, University Hospital Würzburg, since 2021 University Hospital Augsburg, Augsburg, Germany; 20 Diagnostic and Interventional Neuroradiology, Faculty of Medicine, University of Augsburg, Augsburg, Germany; 21 Institute of Human Genetics, Heidelberg University Hospital, Heidelberg, Germany; 22 Institute of Neuropathology, University Hospital Münster, Münster, Germany; 23 Department of Paediatric Haematology and Oncology, University Medical Center Hamburg-Eppendorf, Hamburg, Germany; 24 Research Institute Children’s Cancer Center Hamburg, Hamburg, Germany; 25 Department of Oncology, University Children’s Hospital Zürich, Zürich, Switzerland; 26 Genetics and Molecular Pathology Laboratory, Hudson Institute of Medical Research, Clayton, VIC Australia; 27 Department of Molecular and Translational Science, Monash University, Melbourne, VIC Australia; 28 Victorian Clinical Genetics Services, Parkville, VIC Australia; 29 Australian and New Zealand Children’s Haematology and Oncology Group (ANZCHOG), Hudson Institute of Medical Research, Clayton, VIC Australia; 30 Department of Paediatrics, University of Melbourne, Parkville, VIC Australia; 31 Women’s and Children’s Hospital, South Australia Health and Medical Research Institute, South Australia immunoGENomics Cancer Institute, University of Adelaide, Adelaide, SA Australia; 32 Department of Paediatric and Adolescent Oncology/Haematology, Perth Children’s Hospital, Nedlands, WA Australia; 33 Centre for Child Health Research, University of Western Australia, Nedlands, WA Australia; 34 Brain Tumour Research Program, Telethon Kids Institute, Nedlands, WA Australia; 35 Department of Neurosurgery, University of Tuebingen, Tuebingen, Germany; 36 Department of Neurosurgery, Charité – Universitätsmedizin Berlin, Berlin, Germany; 37 German HIT-LOGGIC Registry for low-grade glioma in children and adolescents, Department of Pediatric Oncology and Hematology, Charité - Universitätsmedizin Berlin, corporate member of Freie Universität Berlin and Humboldt-Universität zu Berlin, Berlin, Germany; 38 Swabian Children’s Cancer Center, Paediatric and Adolescent Medicine, Faculty of Medicine, University Augsburg, Augsburg, Germany; 39 Clinical Cooperation Unit Pediatric Oncology, German Cancer Research Center (DKFZ) and German Cancer Consortium (DKTK), Heidelberg, Germany; 40 Department of Pediatrics and Pediatric Hematology/Oncology, University Children’s Hospital, Klinikum Oldenburg AöR, Oldenburg, Germany; 41 Department of Pediatric Hematology and Oncology, University Childrens’ Hospital Muenster, Muenster, Germany; 42 Pediatric Hematology and Oncology, Pediatrics III, University Children’s Hospital of Essen, Essen, Germany; 43 Mildred Scheel Cancer Career Center HaTriCS4, University Medical Center Hamburg-Eppendorf, Hamburg, Germany; 44 Department of Child and Adolescent Health, Division of Pediatric Hematology and Oncology, University Medical Center Göttingen, Göttingen, Germany

**Keywords:** CNS cancer, Paediatric cancer, Cancer epigenetics, Cancer epidemiology, Diagnostic markers

Correction to: *Nature Medicine* 10.1038/s41591-023-02255-1. Published online 16 March 2023.

In the version of this article initially published, Arend Koch (Department of Neuropathology, Charité - Universitätsmedizin Berlin, Berlin, Germany), Martin U. Schuhmann (Department of Neurosurgery, University of Tuebingen, Tuebingen, Germany) and Ulrich W. Thomale (Department of Neurosurgery Charité – Universitätsmedizin Berlin, Berlin, Germany) were missing from the author list and are now included in the HTML and PDF versions of the article.

