# Supplementary material for: Multiomic neuropathology improves diagnostic accuracy in pediatric neuro-oncology
Source: Nat Med. 2023 Mar 16;29(4):917–26. doi: 10.1038/s41591-023-02255-1 (PMC10115638; doi:10.1038/s41591-023-02255-1)
Supplement: Supplementary file 1 — Supplementary Figs. 1–17 and Supplementary Table legends. [file 41591_2023_2255_MOESM1_ESM.pdf]

nature medicine

Article

https://doi.org/10.1038/s41591-023-02255-1

Multiomic neuropathology improves diagnostic accuracy in pediatric neuro-oncology

In the format provided by the authors and unedited

Multiomic neuropathology improves diagnostic accuracy in pediatric neuro-oncology: Supplementary Information

Table of contents

Supplementary Figures 3

Supplementary Fig. 1 | Patient recruitment by study center and over time 3

Supplementary Fig. 2 | Tumor categories by WHO- and DNA methylation-based classification and epidemiological assessment 4

Supplementary Fig. 3 | Registration and sample processing timelines 5

Supplementary Fig. 4 | Significant regions of DNA copy-number alterations by DNA methylation class 6

Supplementary Fig. 5 | Summary of DNA copy-number alterations by DNA methylation class 9

Supplementary Fig. 6 | Comparison of DNA methylation-based and WHO-based CNS tumor classification 10

Supplementary Fig. 7 | Correlation between DNA methylation-based and WHO-based CNS tumor classification 11

Supplementary Fig. 8 | Landscape of DNA methylation classes (by t-SNE analysis) and levels of concordance with WHO-based tumor type 12

Supplementary Fig. 9 | Landscape of somatic and constitutional alterations per category .. 13

Supplementary Fig. 10 | Molecular risk stratification of pediatric patients with high-grade glioma 15

Supplementary Fig. 11 | Risk stratification of patients with molecularly defined high-grade glioma 16

Supplementary Fig. 12 | Event-free survival of patients low-grade glioma 17

Supplementary Fig. 13 | Overall survival of patients with medulloblastoma 18

Supplementary Fig. 14 | Overall survival of patients with ependymal tumors 19

Supplementary Fig. 15 | Overall survival of patients with other embryonal/pineal tumors . 20

Supplementary Fig. 16 | Distribution of tumors with DNA methylation class prediction scores < 0.9 21

Supplementary Fig. 17 | In silico analyses of tumor cell content 22

Supplementary Table Legends..23

Supplementary Table 1 | CNS tumor classification; clinical patient data; levels of concordance; interdisciplinary tumor board discussions; clinical patient follow-up data; DNA methylation class prediction scores.. 23

Supplementary Table 2 | Significant regions of DNA copy-number alterations by DNA methylation class... 23

Supplementary Table 3 | Pairwise correlation between DNA methylation classes and WHO tumor types.. 23

Supplementary Table 4 | t-distributed stochastic neighbor embedding (t-SNE) 23

Supplementary Table 5 | NGS gene panel 23

Supplementary Table 6 | Genetic alterations detected by NGS from tumor and blood samples.. 24

Supplementary Table 7 | List of predefined cancer predisposing genes............................... 24

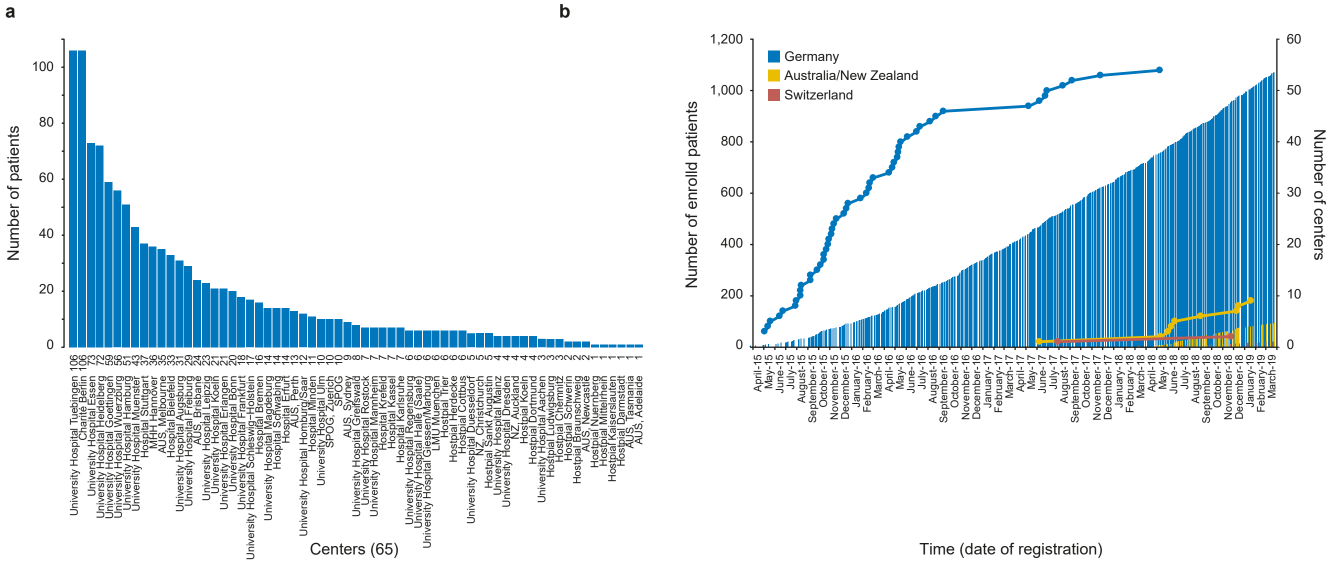

Supplementary Figure 1 | Patient recruitment by study center and over time

a, Patient recruitment by recruiting study center.

b, Patient recruitment (left y-axis) and participating centers (right y-axis) over time.

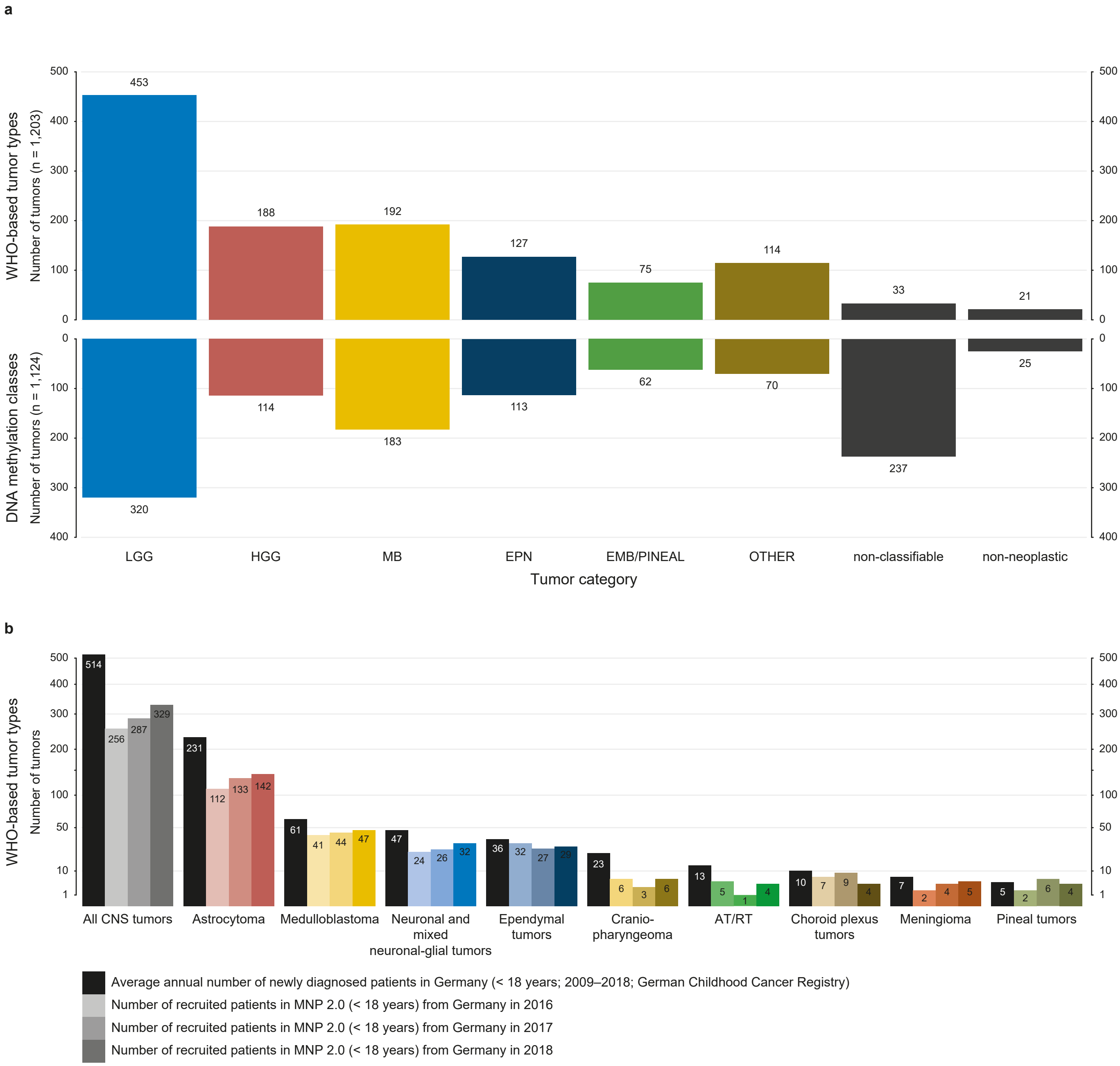

Supplementary Figure 2 | Tumor categories by WHO- and DNA methylation-based classification and epidemiological assessment

a, Tumor classification into WHO-based (upper panel) and DNA methylation-based (lower panel) CNS tumor categories.
b, Comparison of annual patient recruitment (2016–2018) with average numbers of newly diagnosed patients in Germany (2009–2018). Tumors are aligned to epidemiological groups from the German Childhood Cancer Registry[8]. Y-axis scale is square root transformed for improved visibility of tumor groups occurring at low frequency.

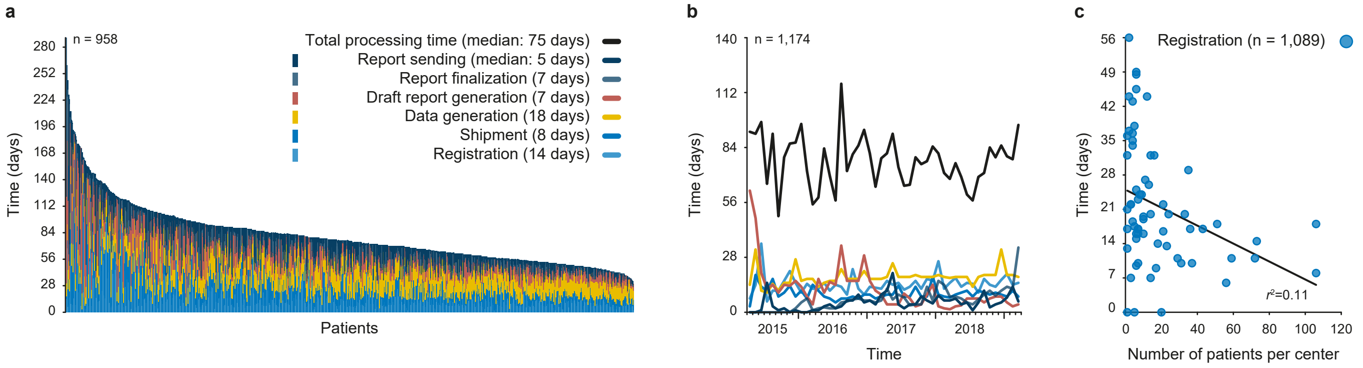

Supplementary Figure 3 | Registration and sample processing timelines

a, Recorded processing times from operation to reporting date for 958 cases for which all steps were individually documented.

b, Processing times corresponding to steps indicated in (**a**) over time.

c, Correlation of median time between operation and patient registration with number of enrolled patients per recruiting center.

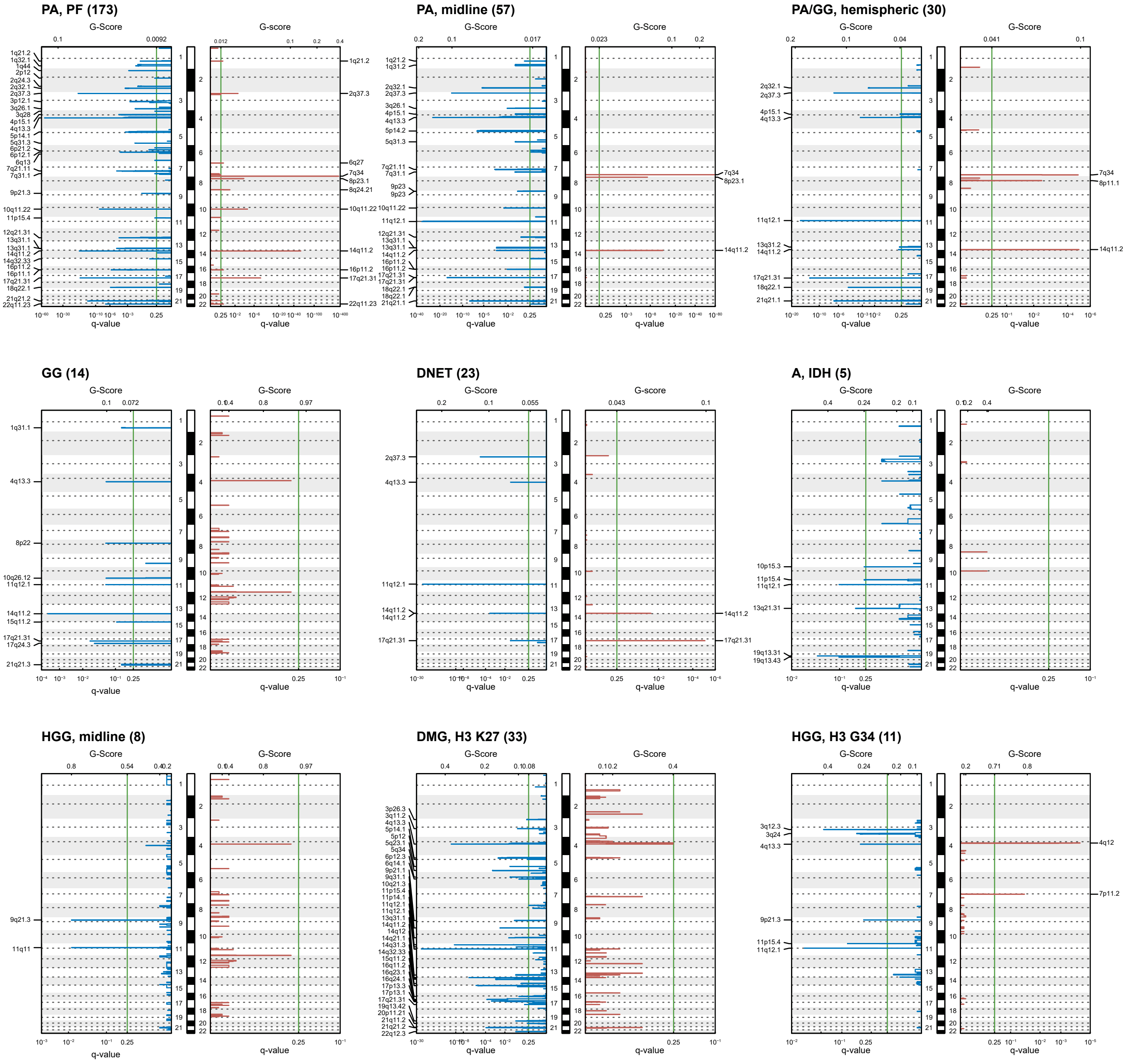

Supplementary Figure 4 | Significant regions of DNA copy-number alterations by DNA methylation class
Plots show the q-values (x-axes, indicating the false discovery rate) determined by GISTIC2.0 with respect to significant lost (blue) and gained (red) genomic regions among the human chromosomes 1 to 22 (hg19) in DNA methylation classes with ≥ five samples. Numbers in brackets indicate sample size for each class. Green lines indicate the significance threshold of q-value < 0.25. The cytobands of significantly altered regions are denoted on the y-axes. See Supplementary Table 2 for a detailed overview of amplified/deleted regions.

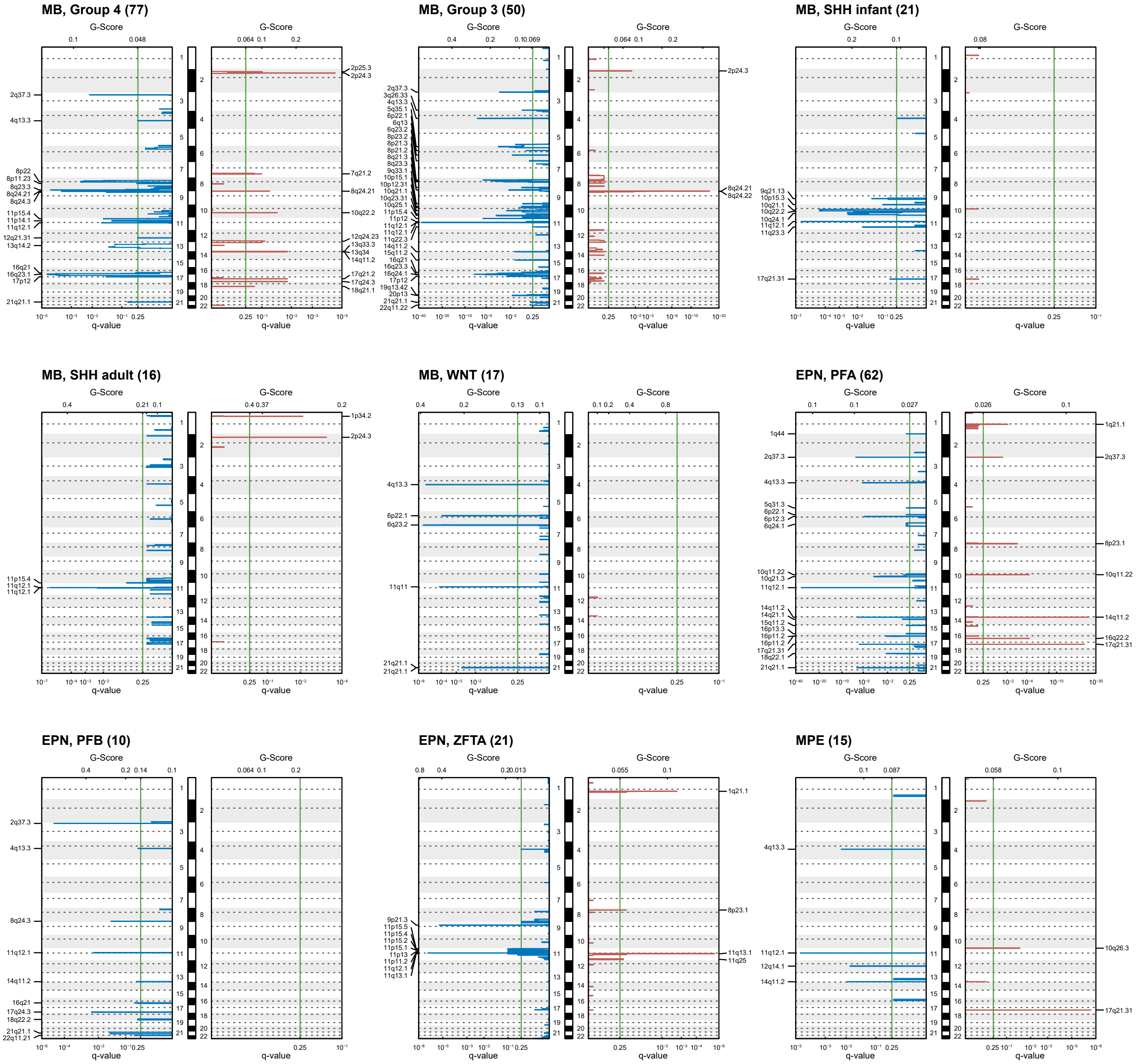

Supplementary Figure 4 (continued) | Significant regions of DNA copy-number alterations by DNA methylation class
Plots show the q-values (x-axes, indicating the false discovery rate) determined by GISTIC2.0 with respect to significant lost (blue) and gained (red) genomic regions among the human chromosomes 1 to 22 (hg19) in DNA methylation classes with ≥ five samples. Numbers in brackets indicate sample size for each class. Green lines indicate the significance threshold of q-value < 0.25. The cytobands of significantly altered regions are denoted on the y-axes. See Supplementary Table 2 for a detailed overview of amplified/deleted regions.

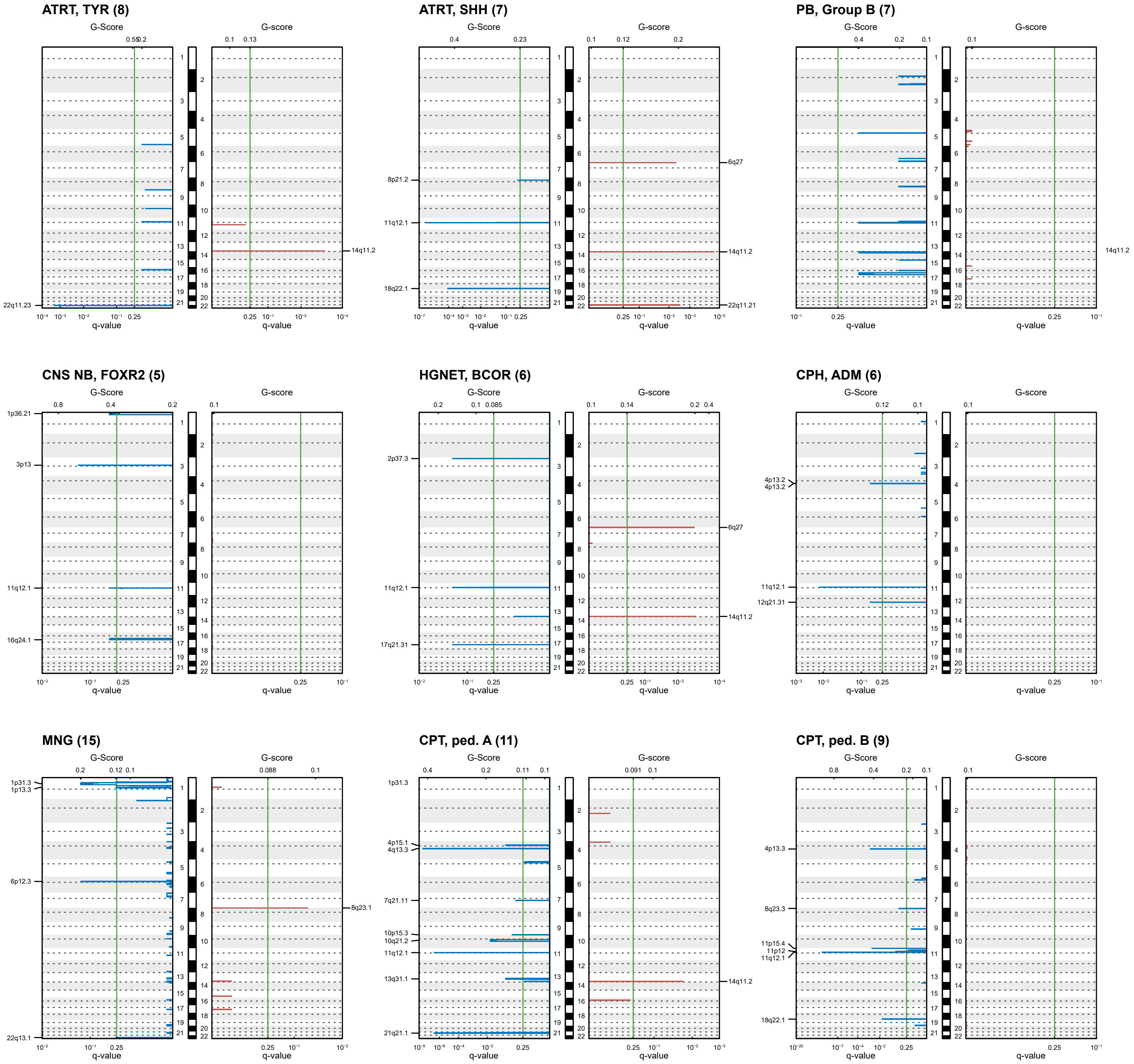

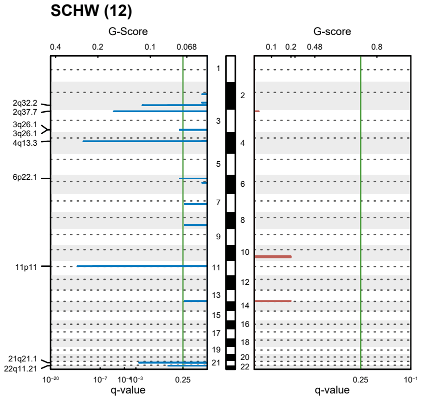

Supplementary Figure 4 (continued) | Significant regions of DNA copy-number alterations by DNA methylation class

Plots show the q-values (x-axes, indicating the false discovery rate) determined by GISTIC2.0 with respect to significant lost (blue) and gained (red) genomic regions among the human chromosomes 1 to 22 (hg19) in DNA methylation classes with ≥ five samples. Numbers in brackets indicate sample size for each class. Green lines indicate the significance threshold of q-value < 0.25. The cytobands of significantly altered regions are denoted on the y-axes. See Supplementary Table 2 for a detailed overview of amplified/deleted regions.

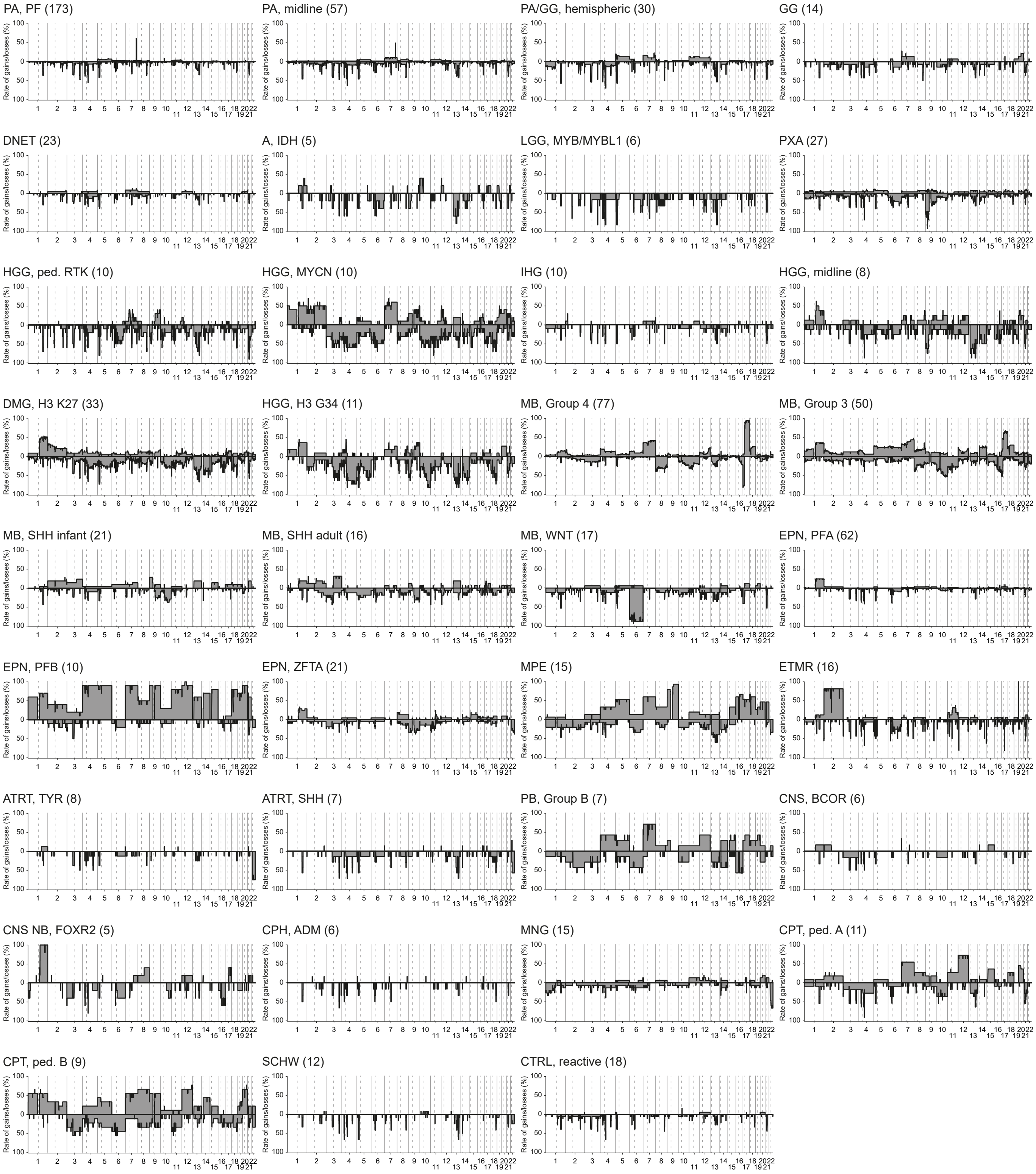

Supplementary Figure 5 | Summary of DNA copy-number alterations by DNA methylation class

Rate of chromosomal gains and losses by DNA methylation class (as depicted in Fig. 1) in DNA methylation classes with ≥ five samples. Each panel depicts one DNA methylation class as labelled. Numbers in brackets indicate sample size for each class.

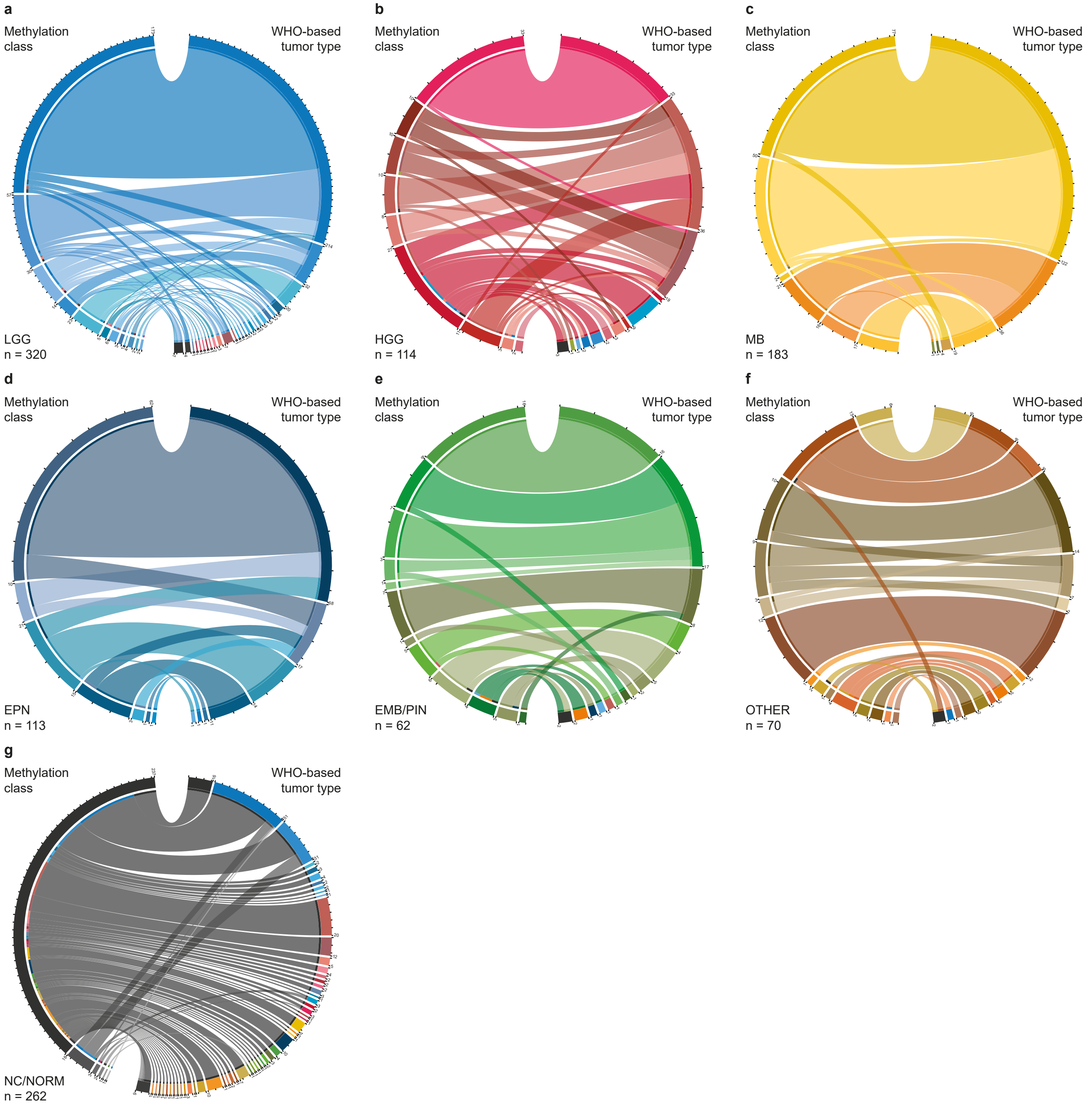

Supplementary Figure 6 | Comparison of DNA methylation-based and WHO-based CNS tumor classification
Comparison of assigned DNA methylation classes (left semicircle) and WHO based diagnoses (right semicircle) across low-grade gliomas (LGG, **a**), high-grade gliomas (HGG, **b**), medulloblastomas (MB, **c**), ependymal tumors (EPN, **d**), embryonal/pineal tumors (EMB/PIN, **e**), other classes (**f**), and samples with a DNA methylation profile matching non-neoplastic control tissue or none of the established reference classes (**g**). Colors correspond to tumor types and classes as indicated in Fig. 1 and Extended Data Fig. 1. Categories in a–g are composed by DNA methylation class; see Fig. 2 and Extended Data Fig. 5 for composition by WHO based tumor type. See Supplementary Table 1 for underlying data.

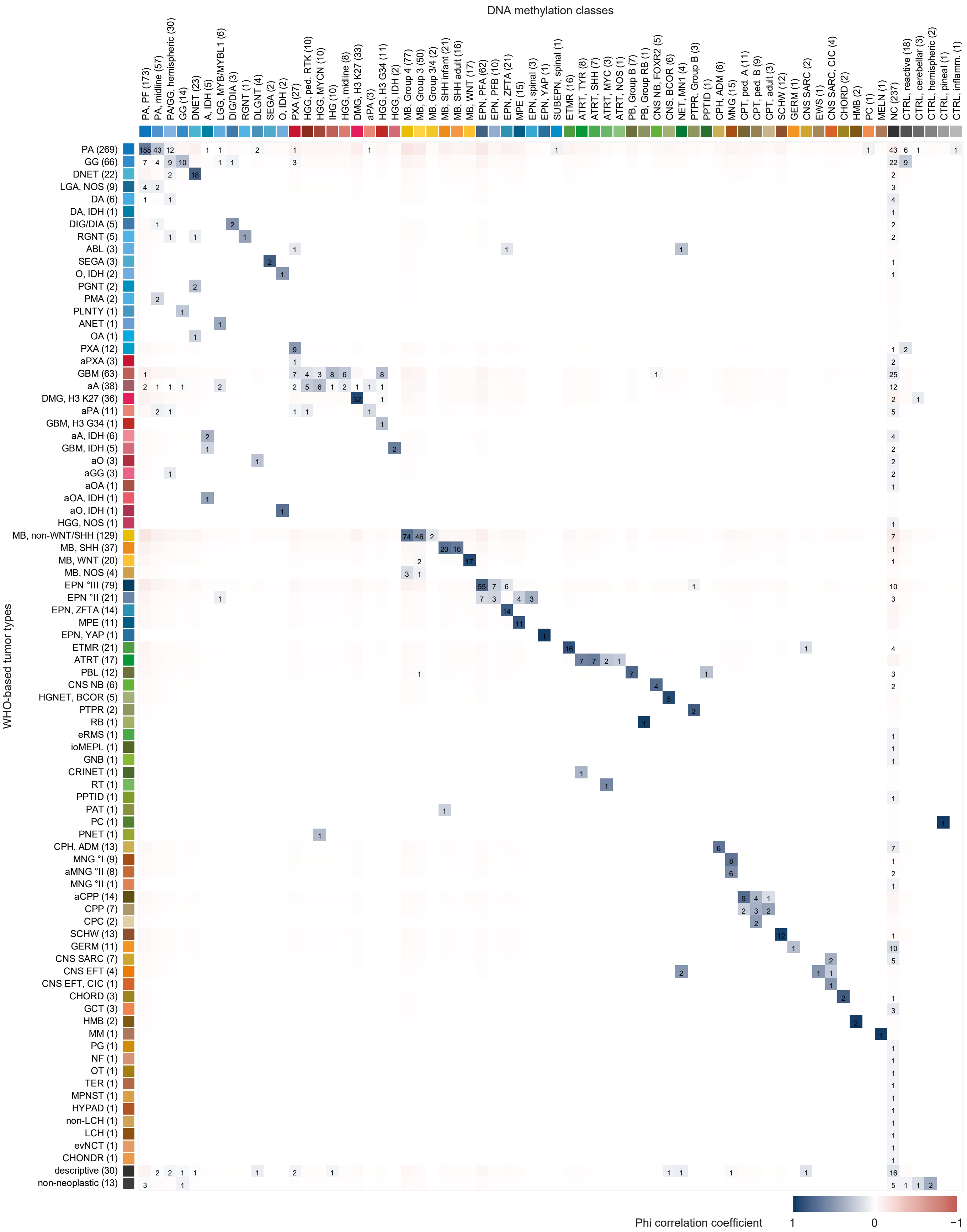

Supplementary Figure 7 | Correlation between DNA methylation-based and WHO-based CNS tumor classification
Correlation between DNA methylation-based and WHO-based CNS tumor classification. Phi correlation coefficient between DNA methylation based classes and WHO based tumor types is represented by a color scale as indicated. Numbers in brackets indicate the number of tumors per tumor type/class. All possible correlations between DNA methylation classes and WHO-based tumor types are displayed. Numbers in tiles indicate the number of tumors per combination. See Supplementary Table 1 and Supplementary Table 3 for underlying data.

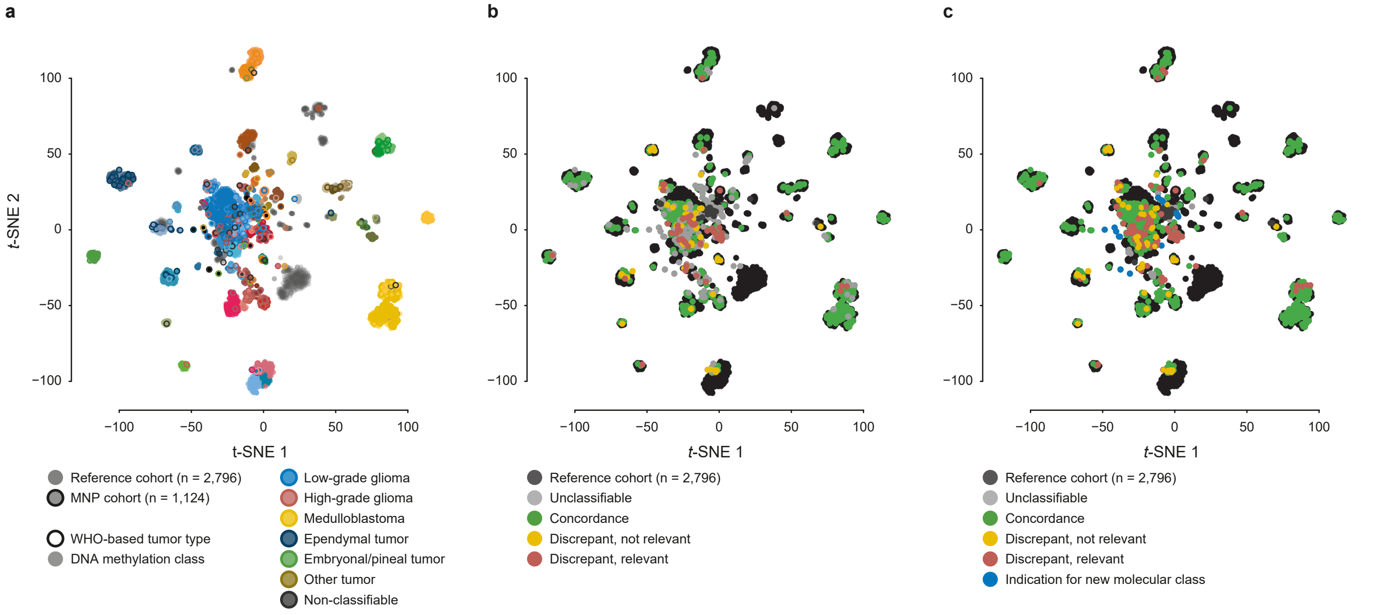

Supplementary Figure 8 | Landscape of DNA methylation classes (by t-SNE analysis) and levels of concordance with WHO-based tumor type

a, *t*-distributed stochastic neighbor embedding (*t*-SNE) analysis of DNA methylation data from the study cohort alongside a reference cohort of 89 published DNA methylation classes[4]. Each tumor from the study cohort is represented by a circle indicating DNA methylation class assigned by visual inspection of *t*-SNE analysis (fill) and WHO-based tumor type (outline). Colors correspond to tumor types and classes as indicated in Fig. 1 and Extended Data Fig. 1.

b and **c**, *t*-SNE analysis corresponding to (**a**), with samples colored according to the level of concordance between WHO-based tumor type and random forest (RF)-based class prediction (**b**) and *t*-SNE-based class assignment (**c**) as depicted in (**a**). See Supplementary Table 1 and Supplementary Table 4 for underlying data.

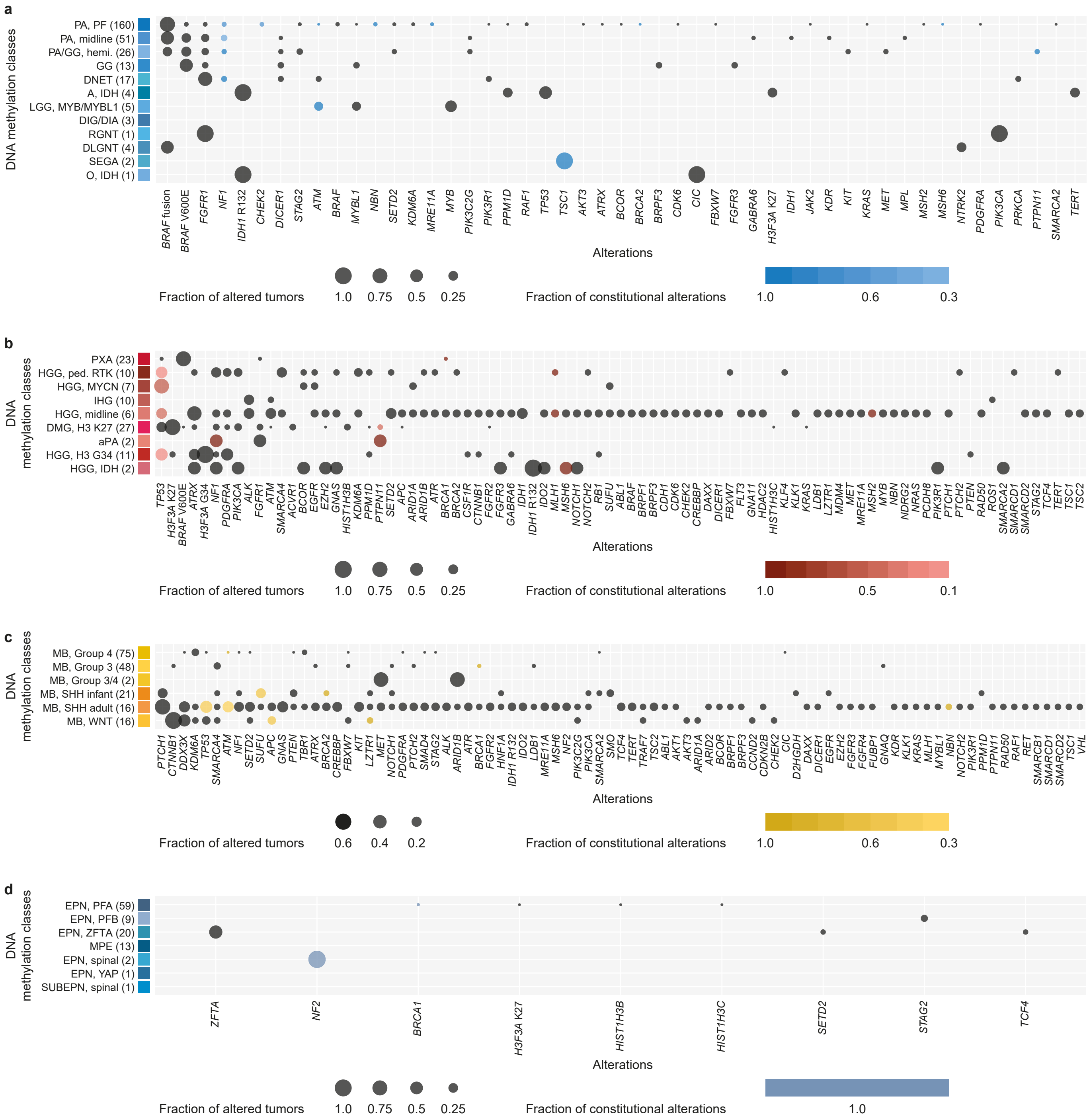

Supplementary Figure 9 | Landscape of somatic and constitutional alterations per category

Frequency of alterations detected in tumors (indicated by circle size) and fraction of alterations detected in corresponding constitutional DNA (color scale) across DNA methylation classes in low-grade gliomas (**a**), high-grade gliomas (**b**), medulloblastomas (**c**), and ependymal tumors (**d**). Gene alterations (x-axis) are ranked by number of affected samples. Numbers in brackets indicate tumors with available sequencing data. See Supplementary Table 6 for underlying data.

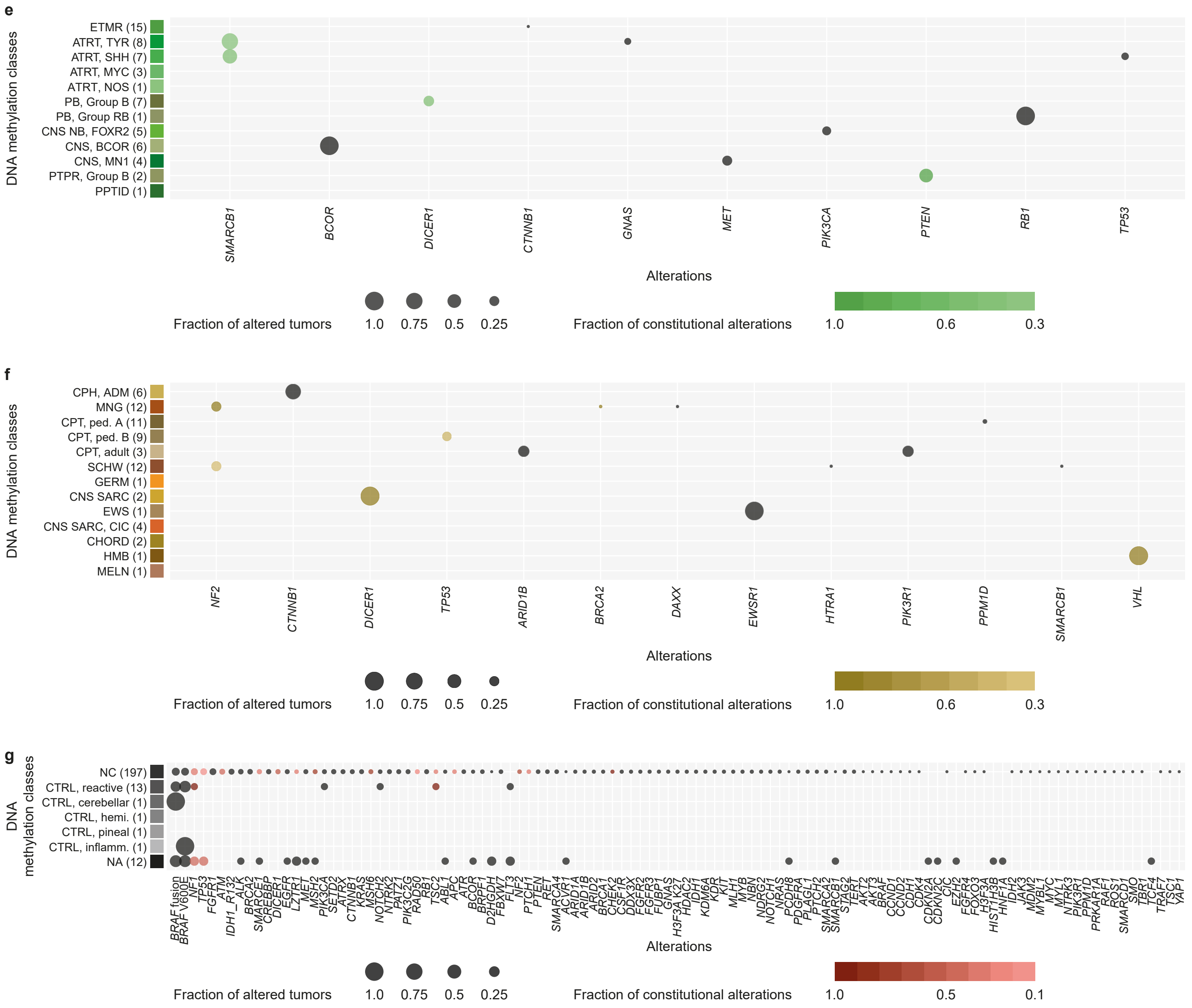

Supplementary Figure 9 (continued) | Landscape of somatic and constitutional alterations per category
Frequency of alterations detected in tumors (indicated by circle size) and fraction of alterations detected in corresponding constitutional DNA (color scale) across DNA methylation classes in embryonal and pineal tumors (**e**), other tumors (**f**), and tumors unclassifiable or assigned to a control class by RF-based DNA methylation class prediction (**g**). Gene alterations (x-axis) are ranked by number of affected samples. Numbers in brackets indicate tumors with available sequencing data. See Supplementary Table 6 for underlying data.

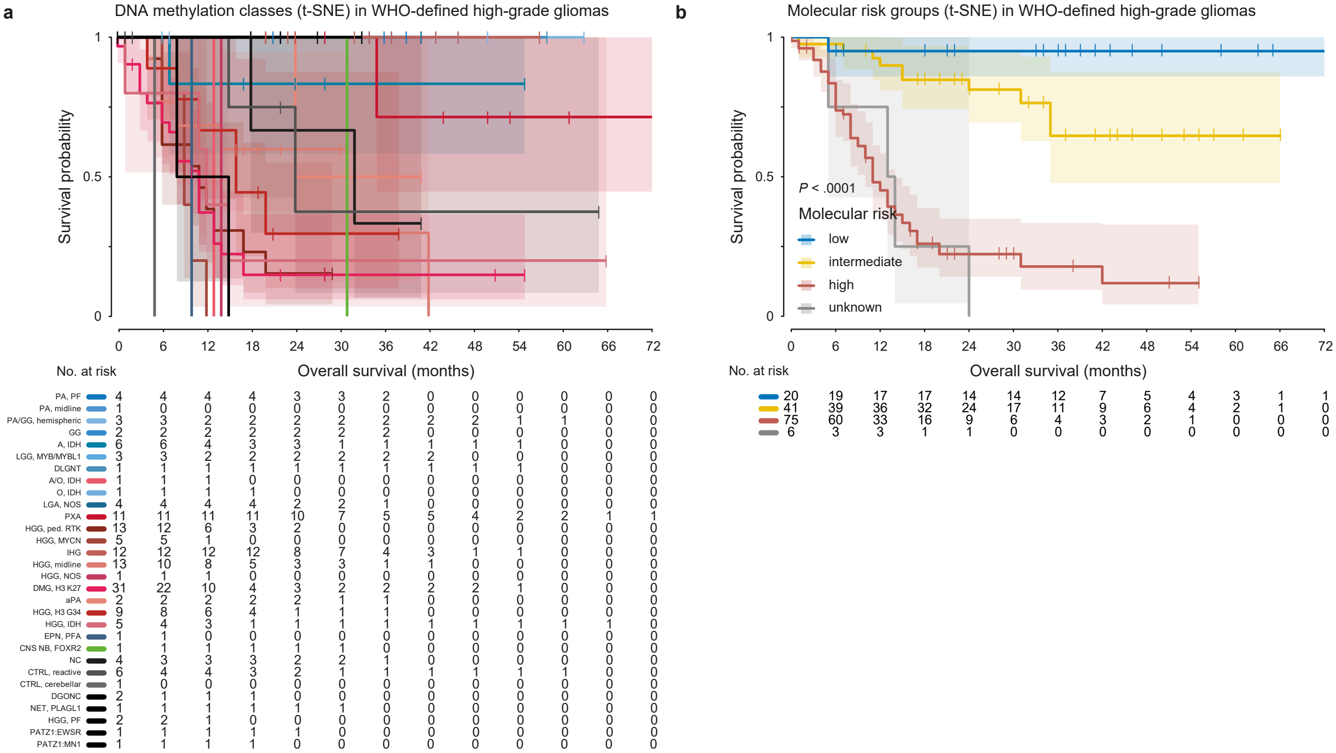

Supplementary Figure 10 | Molecular risk stratification of pediatric patients with high-grade glioma
Kaplan-Meier analysis of overall survival in patients with WHO-defined high-grade gliomas according to DNA methylation class assigned by *t*-SNE analysis (**a**), and molecular risk group (**b**). Colors in (**a**) correspond to tumor types and classes as indicated in Fig. 1 and Extended Data Fig. 1. Shaded areas indicate the 95 % confidence interval for each Kaplan-Meier estimate (solid lines). See Supplementary Table 1 for underlying data.

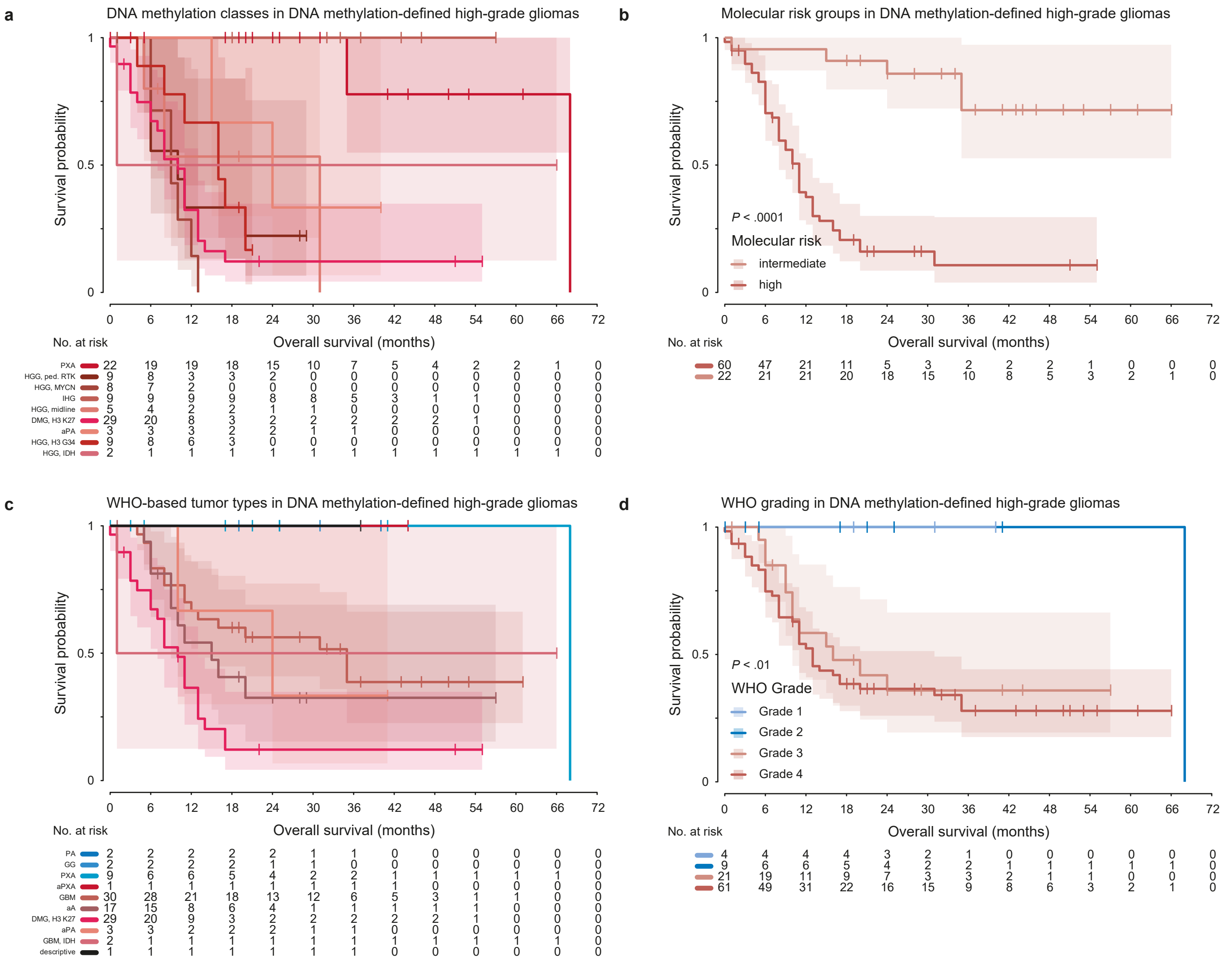

Supplementary Figure 11 | Risk stratification of patients with molecularly defined high-grade glioma
Kaplan-Meier analysis of overall survival in patients with DNA methylation-defined high-grade gliomas according to DNA methylation class (**a**), molecular risk group (**b**), WHO-based tumor type (**c**), and WHO grade (**d**). Colors in (**a**) and (**c**) correspond to tumor types and classes as indicated in Fig. 1 and Extended Data Fig. 1. Shaded areas indicate the 95 % confidence interval for each Kaplan-Meier estimate (solid lines). See Supplementary Table 1 for underlying data.

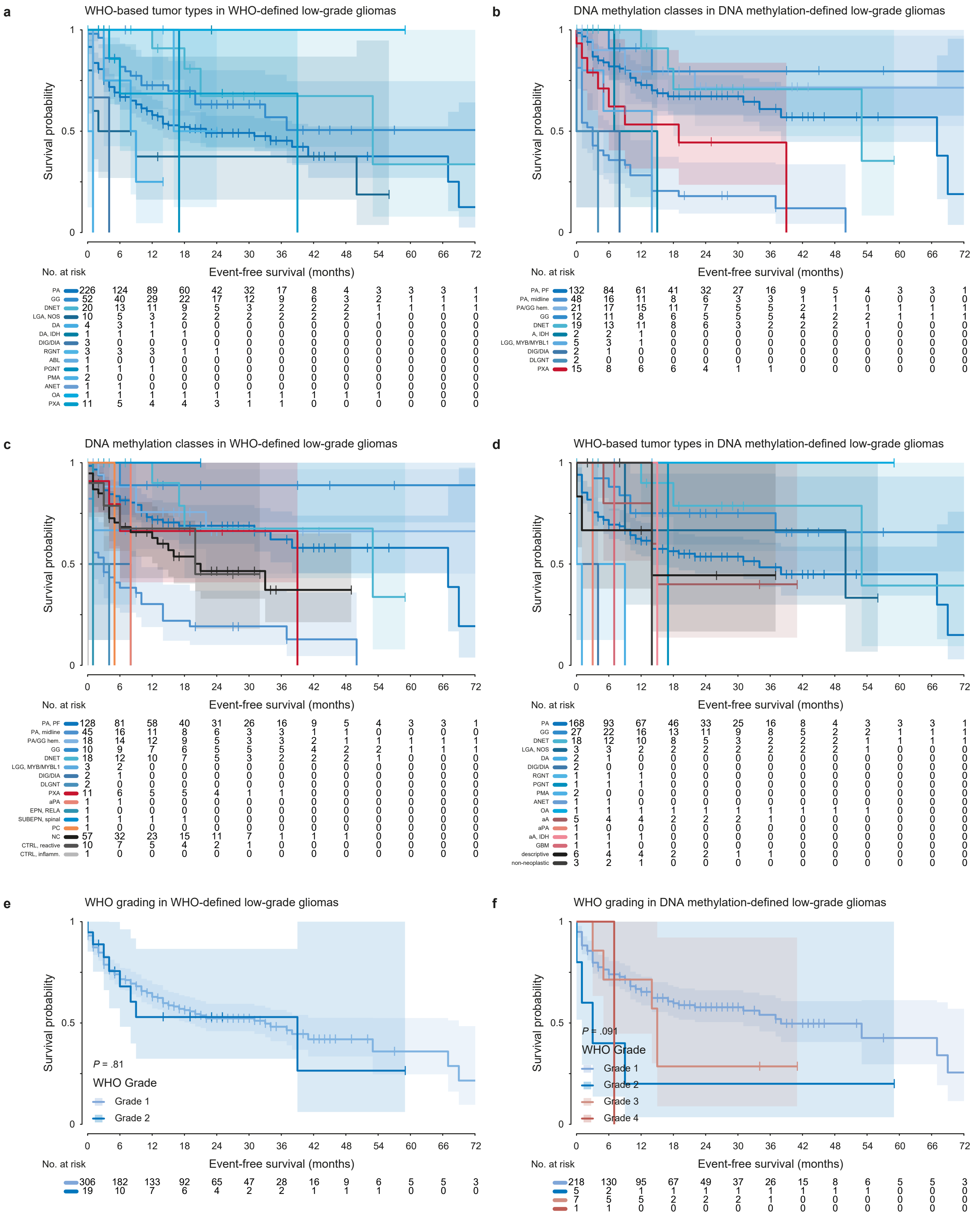

Supplementary Figure 12 | Event-free survival of patients with low-grade glioma

Kaplan-Meier analysis of event-free survival in patients with WHO-defined (**a**,**c**,**e**) and DNA methylation-defined (**b**,**d**,**f**) low-grade gliomas according to WHO-based tumor type (**a**,**d**), DNA methylation class (**b**,**c**), and WHO grade (**e**,**f**). Panel (**b**) includes DNA methylation-defined PXA for comparison. Colors correspond to tumor types and classes as indicated in Fig. 1 and Extended Data Fig. 1. Shaded areas indicate the 95 % confidence interval for each Kaplan-Meier estimate (solid lines). See Supplementary Table 1 for underlying data.

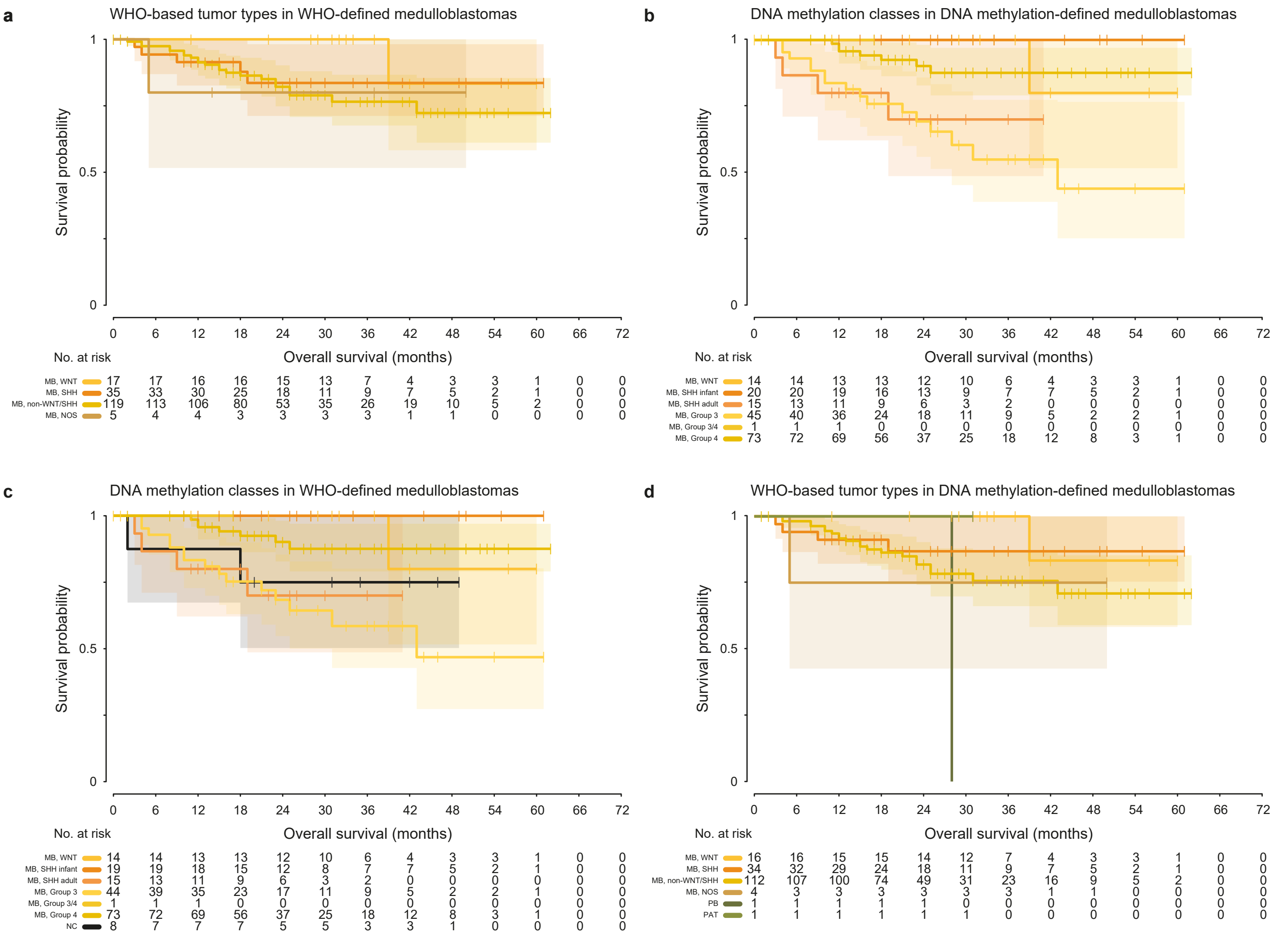

Supplementary Figure 13 | Overall survival of patients with medulloblastoma

Kaplan-Meier analysis of overall survival in patients with WHO-defined (**a**,**c**) and DNA methylation-defined (**b**,**d**) medulloblastomas according to WHO-based tumor type (**a**,**d**) and DNA methylation class (**b**,**c**). Colors correspond to tumor types and classes as indicated in Fig. 1 and Extended Data Fig. 1. Shaded areas indicate the 95 % confidence interval for each Kaplan-Meier estimate (solid lines). See Supplementary Table 1 for underlying data.

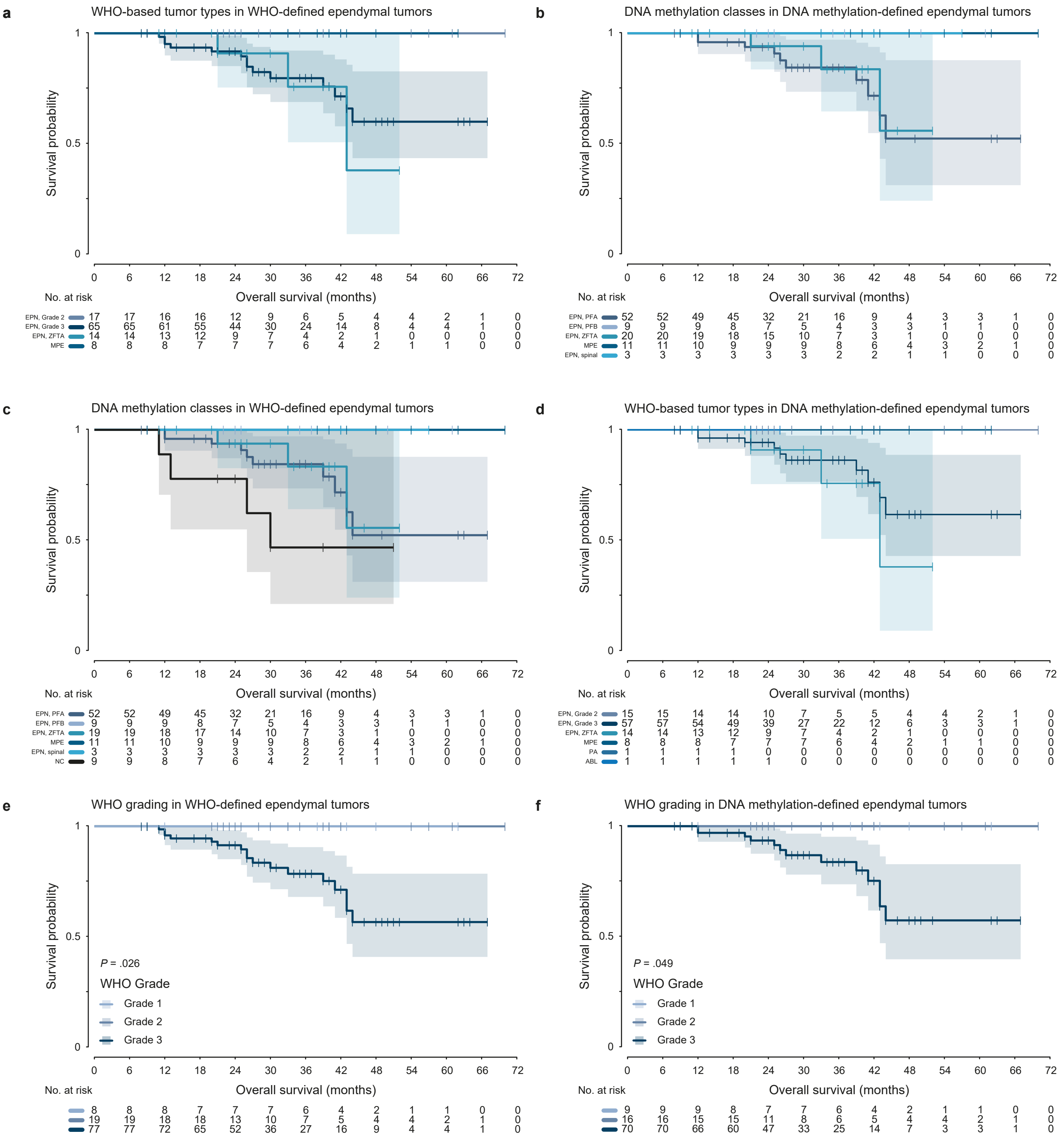

Supplementary Figure 14 | Overall survival of patients with ependymal tumors
Kaplan-Meier analysis of overall survival in patients with WHO-defined (**a**,**c**) and DNA methylation-defined (**b**,**d**) ependymal tumors according to WHO-based tumor type (**a**,**d**), DNA methylation class (**b**,**c**), and WHO grade (**e**,**f**). Colors correspond to tumor types and classes as indicated in Fig. 1 and Extended Data Fig. 1. Shaded areas indicate the 95 % confidence interval for each Kaplan-Meier estimate (solid lines). See Supplementary Table 1 for underlying data.

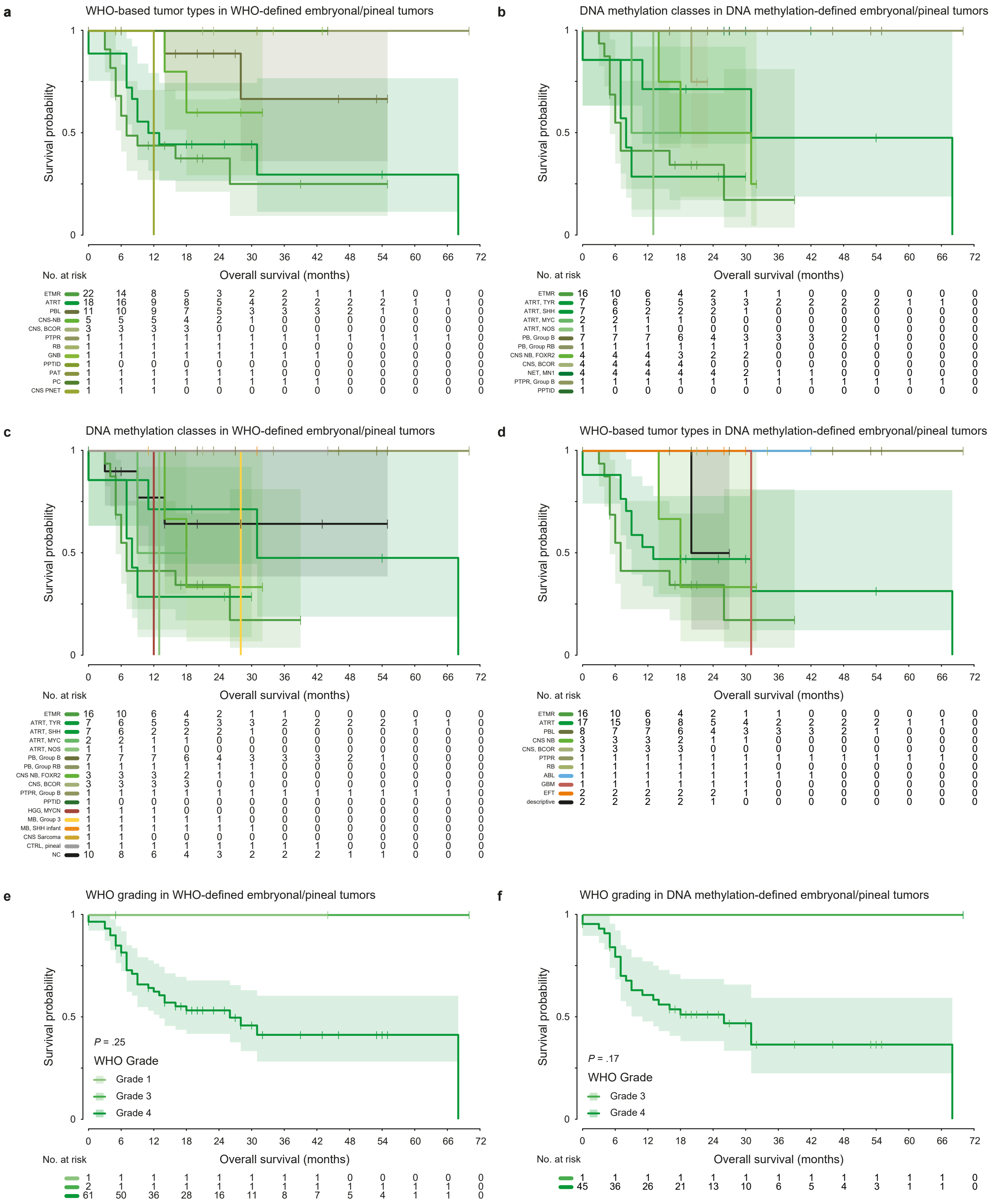

Supplementary Figure 15 | Overall survival of patients with other embryonal/pineal tumors

Kaplan-Meier analysis of overall survival in patients with WHO-defined (**a**,**c**) and DNA methylation-defined (**b**,**d**) embryonal and pineal tumors according to WHO-based tumor type (**a**,**d**), DNA methylation class (**b**,**c**), and WHO grade (**e**,**f**). Colors correspond to tumor types and classes as indicated in Fig. 1 and Extended Data Fig. 1. Shaded areas indicate the 95 % confidence interval for each Kaplan-Meier estimate (solid lines). See Supplementary Table 1 for underlying data.

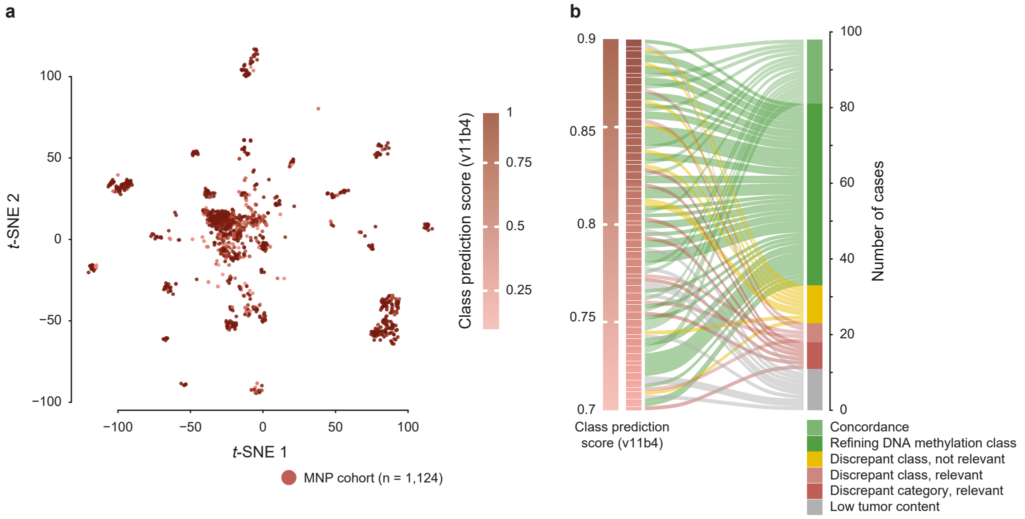

Supplementary Figure 16 | Distribution of tumors with DNA methylation class prediction scores < 0.9

a, *t*-distributed stochastic neighbor embedding (*t*-SNE) analysis of DNA methylation data from the study cohort color-coded by random forest (RF)-based DNA methylation class prediction score (version v11b4). *t*-SNE coordinates correspond to Fig. 3a and Supplementary Fig. 8.

b, Comparison of certainty levels of WHO-based diagnoses and concordance with DNA methylation classes assigned by RF-based class prediction for tumors with DNA methylation class prediction scores between 0.7 and 0.9.

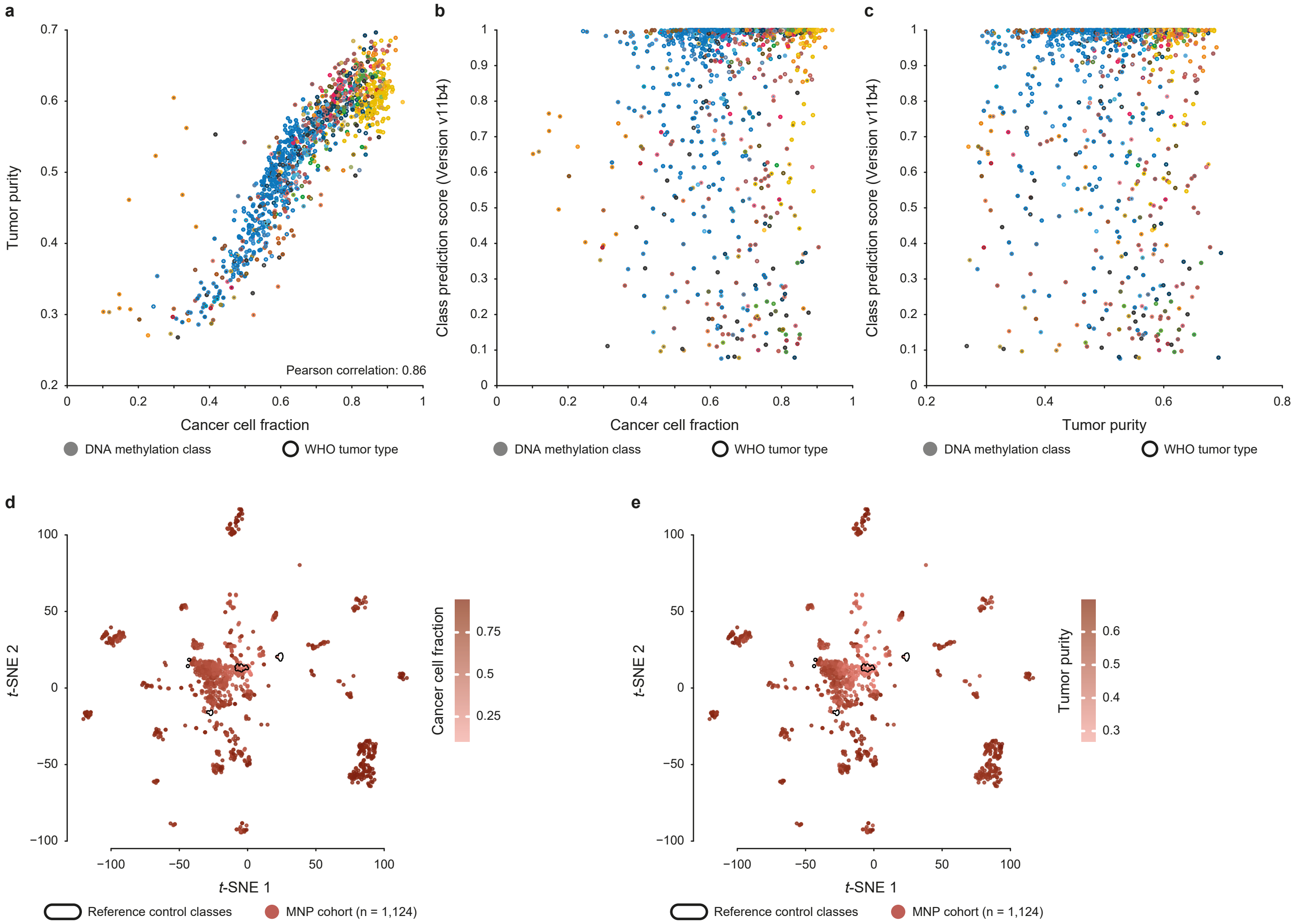

Supplementary Figure 17 | In silico analyses of tumor cell content

a, Correlation of cancer cell fraction and tumor purity predicted in silico using DNA methylation array data (see Online methods). Each tumor from the study cohort is represented by a circle indicating assigned DNA methylation class (fill) and WHO-based tumor type (outline).

b and **c**, Correlation of predicted cancer cell fraction (**b**) and tumor purity (**c**) and DNA methylation class prediction score (using version v11b4 of the random forest class prediction algorithm).

d and **e**, *t* distributed stochastic neighbor embedding (*t*-SNE) analysis of DNA methylation data from the study cohort color-coded by predicted cancer cell fraction (**d**) and tumor purity (**e**). Reference non neoplastic control DNA methylation classes are outlined in black. Colors in (**a**–**c**) correspond to tumor types and classes as indicated in Fig. 1 and Extended Data Fig. 1. *t*-SNE coordinates in (**d**) and (**e**) correspond to Fig. 3a and Supplementary Fig. 8.

SUPPLEMENTARY TABLE LEGENDS

Supplementary Table 1 | CNS tumor classification; clinical patient data; levels of concordance; interdisciplinary tumor board discussions; clinical patient follow-up data; DNA methylation class prediction scores

Classification of 1,204 CNS tumors into WHO-based tumor types, DNA methylation classes, and superordinate categories, as well as available FFPE tissue, generated NGS data and clinical patient data. Levels of concordance (as depicted in Fig. 3c and Supplementary Fig. 8b,c) between WHO- and DNA methylation-based classification of 1,124 CNS tumors. Participants, available data, information, and tumor board consensus (as depicted in Extended Data Fig. 9) for weekly interdisciplinary tumor board discussions. Clinical follow-up data obtained through national study headquarters for 952 patients. Random forest-based DNA methylation class prediction, and calibrated class prediction scores of classifier versions 11b4 and 12.5 for 1,124 CNS tumors (as depicted in Extended Data Fig. 10).

Supplementary Table 2 | Significant regions of DNA copy-number alterations by DNA methylation class

Information on each copy-number alteration peak identified by GISTIC2.0 analysis including genes contained in each peak. Details on amplifications and deletions (per DNA methylation class with ≥ five samples) are listed as separate sheets.

Supplementary Table 3 | Pairwise correlation between DNA methylation classes and WHO tumor types

Phi coefficient and *P*-value for pairwise correlation between DNA methylation classes and WHO tumor types (as depicted in Extended Data Fig. 6).

Supplementary Table 4 |* t*-distributed stochastic neighbor embedding (*t*-SNE)

Annotation and *t*-SNE coordinates of samples from the study cohort (n = 1,124) and reference DNA methylation classes (89; n = 2,796) included in the *t*-SNE analysis (as depicted in Fig. 3 and Supplementary Fig. 8).

Supplementary Table 5 | NGS gene panel

List of 130 gene loci and intronic regions covered by targeted next-generation sequencing.

Supplementary Table 6 | Genetic alterations detected by NGS from tumor and blood samples

Per patient and gene catalogue of somatic and constitutional alterations detected by targeted gene panel sequencing of DNA from tumor tissue and patient blood samples, as well as RNA sequencing of selected tumor samples. Includes classification into WHO-based tumor types, DNA methylation classes, and superordinate categories as indicated in Supplementary Table 1 and information about known cancer predisposition.

Supplementary Table 7 | List of predefined cancer predisposing genes

List of 47 predefined cancer predisposing genes screened by targeted NGS of constitutional DNA.
