# Supplementary material for: Multiomic neuropathology improves diagnostic accuracy in pediatric neuro-oncology
Source: Nat Med. 2023 Mar 16;29(4):917–26. doi: 10.1038/s41591-023-02255-1 (PMC10115638; doi:10.1038/s41591-023-02255-1)
Supplement: Supplementary file 2 — Reporting Summary [file 41591_2023_2255_MOESM2_ESM.pdf]

nature portfolio

Corresponding author(s): David T.W. Jones

Last updated by author(s): Feb 1, 2023

Reporting Summary

Nature Portfolio wishes to improve the reproducibility of the work that we publish. This form provides structure for consistency and transparency in reporting. For further information on Nature Portfolio policies, see our Editorial Policies and the Editorial Policy Checklist.

Statistics

For all statistical analyses, confirm that the following items are present in the figure legend, table legend, main text, or Methods section.

n/a	Confirmed	
☐	☒	The exact sample size (*n*) for each experimental group/condition, given as a discrete number and unit of measurement
☒	☐	A statement on whether measurements were taken from distinct samples or whether the same sample was measured repeatedly
☐	☒	The statistical test(s) used AND whether they are one- or two-sided<br>*Only common tests should be described solely by name; describe more complex techniques in the Methods section.*
☒	☐	A description of all covariates tested
☐	☒	A description of any assumptions or corrections, such as tests of normality and adjustment for multiple comparisons
☐	☒	A full description of the statistical parameters including central tendency (e.g. means) or other basic estimates (e.g. regression coefficient) AND variation (e.g. standard deviation) or associated estimates of uncertainty (e.g. confidence intervals)
☐	☒	For null hypothesis testing, the test statistic (e.g. *F*, *t*, *r*) with confidence intervals, effect sizes, degrees of freedom and *P* value noted<br>*Give P values as exact values whenever suitable.*
☒	☐	For Bayesian analysis, information on the choice of priors and Markov chain Monte Carlo settings
☒	☐	For hierarchical and complex designs, identification of the appropriate level for tests and full reporting of outcomes
☐	☒	Estimates of effect sizes (e.g. Cohen's *d*, Pearson's *r*), indicating how they were calculated

Our web collection on statistics for biologists contains articles on many of the points above.

Software and code

Policy information about availability of computer code

Data collection	No commercial or open source code was used for data collection in this study. Data was collected using a custom database.
Data analysis	DNA methylation-based classification of tumor samples was performed using a random forest (RF) classifying algorithm (available at https://github.com/mwsill/mnp_training) using CNS tumor classifier versions v9.0 , v11.0, v11b2, v11b4, and v12.5 (www.molecularneuropathology.org). Enhanced copy-number variation analysis using Illumina DNA methylation arrays was performed using the R package conumee (versions 1.0.0 to 1.18.0; DOI: 10.18129/B9.bioc.conumee). Summary copy-number plots to display rates of copy-number gains and losses per DNA methylation class were generated using an in house R script (version 1.0; https://github.com/dstichel/CNsummaryplots). GISTIC2.0 (version 2.0.23) analyses were performed to identify genes targeted by somatic copy-number variations per DNA methylation class with a minimum sample size of five via the online platform GenePattern (www.genepattern.org). Tumor location was visualized by adapting the R package cerebroViz (version 1.0; https://github.com/ethanbahl/cerebroViz). Data visualization and statistical analyses were performed using the programming language R (versions 3.6.0 to 4.2.1; https://www.r-project.org/). Palettes of optimally distinct colors were generated and refined using I want hue (version 2.0.0; http://medialab.github.io/iwanthue) and a Graphical User Interface to Pick Colors in HCL Space (http://hclwizard.org:3000/hclcolorpicker).

For manuscripts utilizing custom algorithms or software that are central to the research but not yet described in published literature, software must be made available to editors and reviewers. We strongly encourage code deposition in a community repository (e.g. GitHub). See the Nature Portfolio guidelines for submitting code & software for further information.

Data

Policy information about availability of data

All manuscripts must include a data availability statement. This statement should provide the following information, where applicable:

- Accession codes, unique identifiers, or web links for publicly available datasets
- A description of any restrictions on data availability
- For clinical datasets or third party data, please ensure that the statement adheres to our policy

DNA methylation data generated during this study has been deposited in NCBIs Gene Expression Omnibus (GEO, http://www.ncbi.nlm.nih.gov/geo) under accession number GSE215240. DNA methylation data used as a reference has been deposited under accession number GSE90496. Targeted next-generation DNA sequencing data has been deposited at the European Genome-phenome Archive (EGA, http://www.ebi.ac.uk/ega/) under accession number EGAS00001006680. All source data to replicate our results are provided within Supplementary tables.

Human research participants

Policy information about studies involving human research participants and Sex and Gender in Research.

Reporting on sex and gender	Patient sex and/or gender were not considered in the design of the study, and patient sex was not considered in the inclusion criteria. Patient sex was determined by physical examination by the treating physician responsible for patient registration. No disaggregated information on patient sex and gender was collected in this study.
Population characteristics	Patients were recruited between April 2015 and March 2019 from childhood cancer centers cooperating within the German Society for Pediatric Oncology/Hematology (GPOH), the Swiss Paediatric Oncology Group (SPOG), and the Australian & New Zealand Children's Haematology/Oncology Group (ANZCHOG). Inclusion criteria comprised age ≤ 21 years at primary diagnosis of a CNS neoplasm.
Recruitment	Patients were recruited by local treating pediatric oncologists after consultation of their parents/advocates. Informed consent from adult patients or parental consent was obtained for all patients prior to enrollment. We cannot exclude a self-selection bias towards participants from families with an interest in or supportive of scientific research but consider it highly unlikely to impact our results. Participants were not compensated for their participation.
Ethics oversight	Ethics committee of the medical faculty Heidelberg

Note that full information on the approval of the study protocol must also be provided in the manuscript.

Field-specific reporting

Please select the one below that is the best fit for your research. If you are not sure, read the appropriate sections before making your selection.

☒ Life sciences ☐ Behavioural & social sciences ☐ Ecological, evolutionary & environmental sciences

For a reference copy of the document with all sections, see nature.com/documents/nr-reporting-summary-flat.pdf

Life sciences study design

All studies must disclose on these points even when the disclosure is negative.

Sample size	No sample-size calculation was performed. A total of > 1,000 participants was considered sufficient to identify CNS tumor types occurring at a frequency lower than 1%.
Data exclusions	163 patients that did not fulfill the inclusion criteria (117 recurrences, 23 retrospective registrations, 12 metastases, 11 adults) were excluded from the study.
Replication	Technical robustness of the random forest (RF) classifying algorithm was investigated by inter-laboratory comparison. Results of two independent laboratories (starting from DNA extraction) were compared, and all attempts at replication were successful. See Capper et al. (DOI:10.1038/nature26000) for details.
Randomization	There were no experimental groups or randomization in the study design.
Blinding	As there was no group allocation, blinding of participants was not relevant to our study. Neuropathologists performing reference neuropathological evaluation and neuropathologists as well as scientists perfoming molecular analyses were blinded to the respective results until all analyses were completed.

Reporting for specific materials, systems and methods

We require information from authors about some types of materials, experimental systems and methods used in many studies. Here, indicate whether each material, system or method listed is relevant to your study. If you are not sure if a list item applies to your research, read the appropriate section before selecting a response.

Materials & experimental systems

n/a	Involved in the study
☒	☐ Antibodies
☒	☐ Eukaryotic cell lines
☒	☐ Palaeontology and archaeology
☒	☐ Animals and other organisms
☒	☐ Clinical data
☒	☐ Dual use research of concern

Methods

n/a	Involved in the study
☒	☐ ChIP-seq
☒	☐ Flow cytometry
☒	☐ MRI-based neuroimaging
